# HBP1-mediated transcriptional repression of AFP inhibits hepatoma progression

**DOI:** 10.1186/s13046-021-01881-2

**Published:** 2021-04-01

**Authors:** Zhengyi Cao, Yuning Cheng, Jiyin Wang, Yujuan Liu, Ruixiang Yang, Wei Jiang, Hui Li, Xiaowei Zhang

**Affiliations:** 1 Department of Biochemistry and Biophysics, School of Basic Medical Sciences, Beijing Key Laboratory of Protein Posttranslational Modifications and Cell Function, Peking University Health Science Center, Xueyuan Road 38, Beijing, 100191 People’s Republic of China; 2 Department of Hematology, China-Japan Friendship Hospital, Yinghua East Street, Beijing, 100029 People’s Republic of China

**Keywords:** HBP1, AFP, Transrepression, HBV, Hepatoma

## Abstract

**Background:**

Hepatoma is a common malignancy of the liver. The abnormal high expression of alpha-fetoprotein (AFP) is intimately associated with hepatoma progress, but the mechanism of transcriptional regulation and singularly activation of AFP gene in hepatoma is not clear.

**Methods:**

The expression of transcription factor HBP1 and AFP and clinical significance were further analyzed in hepatoma tissues from the patients who received surgery or TACE and then monitored for relapse for up 10 years. HBP1-mediated transcriptional regulation of AFP was analyzed by Western blotting, Luciferase assay, Realtime-PCR, ChIP and EMSA. After verified the axis of HBP-AFP, its impact on hepatoma was measured by MTT, Transwell and FACS in hepatoma cells and by tumorigenesis in HBP1^−/−^ mice.

**Results:**

The relative expressions of HBP1 and AFP correlated with survival and prognosis in hepatoma patients. HBP1 repressed the expression of AFP gene by directly binding to the AFP gene promoter. Hepatitis B Virus (HBV)-encoded protein HBx promoted malignancy in hepatoma cells through binding to HBP1 directly. Icaritin, an active ingredient of Chinese herb epimedium, inhibited malignancy in hepatoma cells through enhancing HBP1 transrepression of AFP. The repression of AFP by HBP1 attenuated AFP effect on PTEN, MMP9 and caspase-3, thus inhibited proliferation and migration, and induced apoptosis in hepatoma cells. The deregulation of AFP by HBP1 contributed to hepatoma progression in mice.

**Conclusions:**

Our data clarify the mechanism of HBP1 in inhibiting the expression of AFP and its suppression in malignancy of hepatoma cells, providing a more comprehensive theoretical basis and potential solutions for the diagnosis and treatment of hepatoma.

**Supplementary Information:**

The online version contains supplementary material available at 10.1186/s13046-021-01881-2.

## Background

Hepatoma is the fifth largest cancer species in the world, over half a million new cases are diagnosed each year. Due to the poor prognosis, hepatoma is an important cause of cancer-related deaths, with an estimated 695,900 deaths per year [1, 2]. More than 90% of hepatoma is developed on the basis of chronic hepatitis, and the main cause is Hepatitis B Virus (HBV) or Hepatitis C Virus (HCV) infection [3, 4]. Hepatoma usually has no obvious symptoms at an early stage, so nearly 85% of patients are already in intermediate or advanced stage when they are diagnosed [5, 6].

Alpha-fetoprotein (AFP), a glycoprotein, is the most commonly used serum marker for the diagnosis of hepatoma in clinical practice and is widely used in screening, diagnosis, efficacy evaluation and recurrence assessment of hepatoma [7]. Many studies have demonstrated that AFP is an important intracellular signaling molecule involved in a variety of cellular processes, serving as a pro-cancer factor. Li et al. reported that the interaction of AFP and PTEN prohibits the inhibition function of PTEN on the PI3K/AKT pathway, leading to abnormal proliferation of hepatoma cells [8]. It is also reported that the interaction of AFP and caspase-3 blocks the caspase-3 signaling, thereby inhibiting the apoptotic signaling pathway [9]. Furthermore, AFP competitively binds to retinoid acid receptor (RAR), inhibiting the formation of ATRA-RAR complex and nuclear translocation of RAR, up-regulating the expression of survivin, down-regulating the expression of GADD153, Fn14 and GADD45a, resulting in the decrease of cell apoptosis [10–12]. AFP can also promote hepatoma cell migration and invasion by increasing the expression of cell migration-related genes such as K19, EpCAM, MMP2/9 and CXCR4 [13, 14]. These data indicate that AFP interferes with normal signaling networks and leads to tumorigenesis by binding to different signaling proteins. The abnormal high expression of AFP is intimately associated with hepatoma progress, but the mechanism of transcriptional regulation and singularly activation of AFP gene in hepatoma cells is not clear.

The transcription factor HBP1 belongs to the sequence-specific high mobility group (HMG) family and functions as a tumor suppressor. As a transcription factor, HBP1 inhibits its target genes by directly binding to specific affinity elements, such as N-MYC, C-MYC, p47phox, DNMT1 and EZH2 [15–19], all of which are oncogenes or genes that promote tumor development. In addition, HBP1 also transcriptionally activates several downstream genes, including genes encoding p16, p21, myeloperoxidase (MPO) and histone H1 [20–23]. In addition, multiple amino acid residues of HBP1 protein have different chemical modifications. HBP1 can be phosphorylated by p38 and Pim-1 [24, 25], and acetylated by p300/CBP [26], while GID/CTLH and MDM2 can ubiquitinate HBP1 [27, 28]. The type and position of modifications affect the protein stability and transcriptional activity of HBP1. Since HBP1 is involved in the regulation of many important cell cycle regulators, the expression level of HBP1 in cells or tissues affects cell growth or tumorigenesis.

In this study, we found a transcriptional regulatory mechanism to regulate the progression of hepatoma between HBP1 and AFP. We noticed that HBP1 repressed the AFP promoter in a sequence-specific manner through a high affinity site. The repression of AFP by HBP1 inhibited the malignancy of hepatoma cells. In addition, we constructed HBP1-deficient mice and induced hepatoma model by DEN/CCl_4_. Compared to wild-type mice, HBP1-deficient mice developed more severe liver damage, liver fibrosis, and ultimately induced hepatoma with higher malignancy. Since HBP1 and AFP play a key role in the development of hepatoma, studies on the regulation of HBP1 and AFP are crucial in maintaining the normal physiological function of the liver and the diagnosis and treatment of liver cirrhosis and hepatoma.

## Methods

### Cell culture, transfection, and lentivirus

HepG2, and PLC5 (PLC/PRF/5) cells were purchased from the National Institute of Biological Products, Beijing, China. All the cells were cultured in DMEM containing 10% fetal bovine serum. The plasmids were transiently transfected into cells utilizing turbofect transfection reagent (Thermo Scientific) according to the manufacturer’s instruction. The transfection efficiency was detected by western blotting after 48 h post-transfection. The lentivirus plasmid pLL3.7-shHBP1 expresses shRNA that targets HBP1 mRNA (5′-ACTGTGAGTGCCACTTCTC-3′), pLL3.7-shAFP could express shRNA that targets AFP mRNA (5′-GAACGTGGTCAATGTATAA-3′).

### Western blotting and antibodies

Cells were lysed in RIPA buffer (Thermo Scientific) containing protease inhibitor cocktail (Sigma), and BCA protein analysis kit (Pierce) was used to measure the protein concentration. Twenty to Sixty μg of total protein was separated by 8–12% SDS-PAGE and transferred to nitrocellulose membranes (Pall). These primary antibodies we used as follow: anti-HBP1 (11746–1-AP, Proteintech), anti-AFP (14550–1-AP, Proteintech), anti-p-AKT (#4060, Cell Signaling Technology), anti-AKT (#9272, Cell Signaling Technology), anti-MMP9 (10375–2-AP, Proteintech), anti-Csapase-3 (#14220, Cell Signaling Technology), anti-Type I (14695–1-AP, Proteintech), anti-Type III (22734–1-AP, Proteintech), anti-IL-1β (sc-52,012, Santa Cruz), anti-TNF-α (17590–1-AP, Proteintech), anti-FLAG (F1804, Sigma-Aldrich), anti-HA (MMS101P, Covance), and anti-GAPDH (KM9002, Sungene). And these secondary antibodies we used as follow: anti-mouse IgG antibody DyLight 800-conjugated (610–145-121, Rockland) and anti-rabbit IgG DyLight 800-conjugated (611–145-002, Rockland). Odyssey infrared imaging system (LI-COR Bioscience, Lincoln, NE) was used to acquire the infrared fluorescence image.

### Realtime PCR

Total RNA was extracted utilizing the RNAsimple Total RNA kit (Tiangen). The ReverAid FirstStrand cDNA Synthesis kit (Thermo Scientific) was used to synthesize cDNA. The mRNA expression level was analyzed by real-time quantitative PCR instrument (Applied Biosystems) with Maxima SYBR Green qPCR Master Mix (Thermo Scientific). The primers’ sequences were shown as follows: human AFP, 5′-GCAGTGAATCTACAGGGACGC-3′ and 5′-ATCCTGATCCAACCAATCACC-3′; human HBP1, 5′-TGAAGGCTGTGATAATGAGGAAGAT-3′ and 5′-CATAGAAAGGGTGGTCCAGCTTA-3′; and human GAPDH, 5′-CCATGGAGAAGGCTGGGG-3′ and 5′-CAAAGTTGTCATGGATGACC-3′; mouse HBP1, 5′-TGGGAAGTGAAGACAAAT-3′ and 5′-TGACAGGGAGGACATACA-3′; mouse AFP, 5′-AAACCTCCAGGCAACAACCA-3′ and 5′-ACTCCAGCGAGTTTCCTTGG-3′; mouse IL-1β, 5′-GAAATGCCACCTTTTGACAGTG-3′ and 5′-TGGATGCTCTCATCAGGACAG-3′; mouse TNF-α, 5′-CAGGCGGTGCCTATGTCTC-3′ and 5′-CGATCACCCCGAAGTTCAGTAG-3′; mouse type I collagen, 5′-GCTCCTCTTAGGGGCCACT-3′ and 5′-CCACGTCTCACCATTGGGG-3′; mouse type III collagen, 5′-ACGTAGATGAATTGGGATGCAG-3′ and 5′-GGGTTGGGGCAGTCTAGTG-3′; mouse β-actin, 5′-AGCCATGTACGTAGCCATCC-3′ and 5′-GCTGTGGTGGTGAAGCTGTA-3′.

### Chromatin immunoprecipitation (ChIP)

The ChIP assays were performed as described previously [19]. The primers’ sequences for AFP promoter were 5′-ACCTCTTTCTGGAGAGTACG-3′ and 5′-GCAGTCATCGTGAAGGTCGT-3′.

### Electrophoretic mobility shift assay (EMSA)

The EMSA assay was performed as described previously [19]. The probes’ sequences were as follow:

AFP-WT:5′-TGGACAAAAACTAACAAATGAATGGGAATTGTACTTGATTAGCAT-3′ and 5′-ATGCTAATCAAGTACAATTCCCATTCATTTGTTAGTTTTTGTCCA-3′.

AFP-MT:5′-TGGACAAAAACTAACAAATGCCTGGGAATTGTACTTGATTAGCAT-3′ and 5′-ATGCTAATCAAGTACAATTCCCAGGCATTTGTTAGTTTTTGTCCA-3′.

### Reporter gene assay

The plasmids were transfected into 293 T cells for individual experiments. Cell lysates were prepared at 24 h after transfection utilizing the Dual Luciferase Reporter Assay Kit (Promega). The Firefly luciferase activity measurement was standardized to the same sample’s Renilla luciferase activity.

### Immunoprecipitation

The plasmids were transfected into 293 T cells for individual experiments. Cells were harvested and lysed in IP lysis buffer (25 mM Tris-HCl (pH 7.4), 150 mM NaCl, 1% Nonidet P-40, 1 mM EDTA, and 5% glycerol) containing protease inhibitor cocktail. The lysates were incubated with primary antibodies and protein A-Sepharose (GE Healthcare) with constant rotation overnight at 4 °C. The samples were washed with IP lysis buffer three times and the relevant protein binding was detected by Western blotting analysis.

### In vivo ubiquitination assay

Two hundred ninety-three T cells transfected with HA-HBP1 were treated with or without Icaritin for 6 h. We harvested and lysed cells by IP lysis buffer. The lysates were incubated with anti-HA antibody and protein A-Sepharose (GE Healthcare). Western blotting was performed with the indicated antibodies.

### MTT assay

HepG2 cell sublines were constructed by lentivirus infection. Cells were seeded into 96-well plates 1000 cells per well. Fifteen μl of MTT solution (5 mg/ml) was added to each well after culturing for 1–7 days separately. Four h later we removed the medium and added 200 μl DMSO to each well to dissolve the formazan crystals. We measured the absorbance each sample at 490 nm utilizing the microplate reader.

### Transwell assay

HepG2 cell sublines were constructed by lentivirus infection. 5 × 10^4^ cells were added to each chamber with 100 μl serum-free medium, and 1 ml of serum-containing medium was added to the lower layer. After continuing to culture for 12 h, the cells in the chamber were wiped with a cotton swab and fixed with 4% paraformaldehyde for 30 min. Stain with 0.5% crystal violet stain for 20 min, then rinse with water to remove excess dye solution. The number of cells passing through the membrane was observed under a microscope, and the number of cells in at least 5 fields of view was counted.

### Apoptosis assay

HepG2 cell sublines were constructed by lentivirus infection. For induction of apoptosis, cells were seeded into 6-well-plate and treated with 150 μM H_2_O_2_ for 24 h. The cells were trypsinized, washed, and then stained with FITC-Annexin V (640,906, Biolegend) and propidium iodide (PI) for 10 min in the dark at room temperature. 1 × 10^5^ cells per sample were acquired and analyzed using the FACS Canto II flow cytometer (BD).

### Animals

The experimental animal facility has been accredited by the AAALAC (Association for Assessment and Accreditation of Laboratory Animal Care International). All mouse experiments conformed to the Guide for the Care and Use of Laboratory Animals of the Health Science Center of Peking University. Mice were housed in groups with 12-h dark-light cycles and had free access to food and water. Mice were sacrificed under the isoflurane inhalation and followed by cervical dislocation. All mouse lines were maintained in a C57BL/6 J genetic background. Primer 5′-CAGCCTCTGACCTCTTCTAGT-3′ and 5′-TATGAAGTGAGAGGACAACGT-3′ were used for PCR analysis to confirm the success of HBP1 knockout.

To induce HCC, 8 weeks old HBP1-knockout male mice and their wild-type (WT) littermates were intraperitoneally injected with DEN at a dose of 100 mg/kg per mouse, and 0.9% saline was injected into the control group. Two weeks later, DEN was intraperitoneally injected at a dose of 50 mg/kg per mouse, and the control group was injected with 0.9% saline. Thereafter, the CCl_4_ and the olive oil were mixed at a ratio of 1:4, and the mice were intragastrically administered twice a week, 100 μl each time, and the control group was intragastrically administered with 100 μl of olive oil for 16 weeks. At the same time, the drinking water of the experimental group was changed to 8% ethanol, and the control group was given normal drinking water.

### Tumor tissues and immunohistochemistry staining

Human tissue microarrays of hepatoma patients (Cat No. LVC-1608) were purchased from Shanghai Superbiotek Pharmaceutical Technology Co. Ltd. (Shanghai, China). The information and samples of these patients were collected from 2008 to 2017. The tissue microarray slides were dewaxed, antigen retrieved and incubated with anti-HBP1 or anti-AFP specific antibodies, followed by biotin labeled secondary antibodies and colored by streptavidin-ALP system.

### Statistical analysis

SPSS 17.0 software was used for statistical analysis. The data are expressed as the mean ± SD (standard mean error) of at least three independent experiments. The data in statistical tests were in accordance with normal distribution and the variance was similar. The statistical analysis was conducted by Student’s t test. *, *p* < 0.05, **, *p* < 0.01.

## Results

### HBP1 regulates AFP expression

**Fig. 1:**
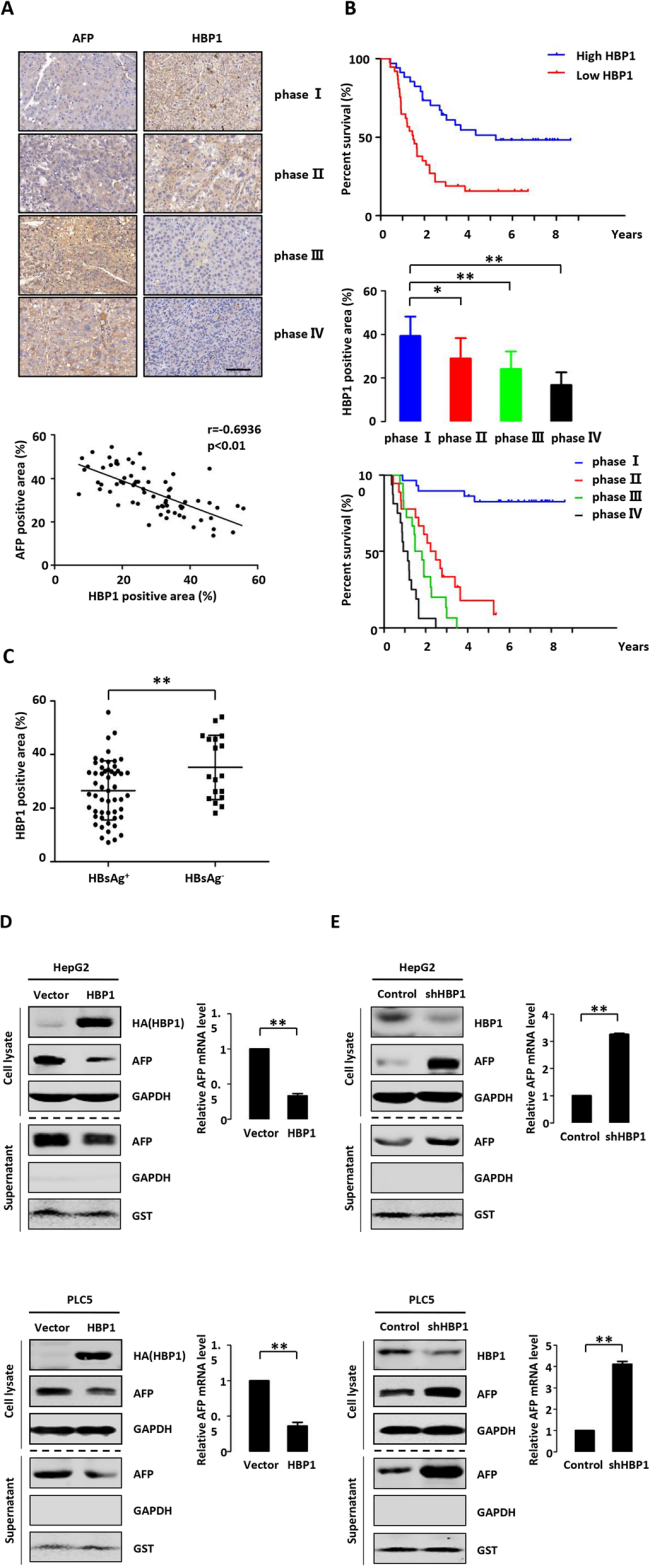
HBP1 regulates AFP expression. **a** There is a negative correlation between the expression of HBP1 and AFP in hepatoma tissues. The gene expression data were derived from the primary tumors who received surgery or TACE and then monitored for relapse for up 10 years. The protein levels of HBP1 and AFP were measured by IHC in hepatoma tissues. Scale bar, 100 μm. **b** Elevated expression of HBP1 in tumor is associated with higher survival rate of hepatoma patients. The overall survival curves were performed in two groups of hepatoma patients with high or low level of HBP1. The protein levels of HBP1 were analyzed based on tumor TNM classification. **c** There is a negative correlation between the expression of HBP1 and HBV infection. The protein level of HBP1 was measured by IHC in hepatoma tissues. **d** HBP1 overexpression decreases AFP protein and mRNA expression. The protein levels of HBP1 and AFP in cell lysate and supernatant were measured by Western blotting in HepG2 and PLC5 cells transfected with pCDNA3-HBP1 or pCDNA3 (as a control). GAPDH and GST were used as a loading control respectively (left panel). The mRNA level of AFP was measured by Realtime-PCR in HepG2 and PLC5 cells transfected with pCDNA3-HBP1 or pCDNA3 (right panel). **e** HBP1 knockdown by shRNA increases AFP protein and mRNA expression. The protein levels of HBP1 and AFP in cell lysate and supernatant were measured by Western blotting in HepG2 and PLC5 cells stably transfected with pLL3.7-shHBP1 or pLL3.7 (as a control) through lentiviral infection. GAPDH and GST were used as a loading control respectively (left panel). The mRNA level of AFP was measured by Realtime-PCR in HepG2 and PLC5 cells stably transfected with pLL3.7-shHBP1 or pLL3.7 (right panel) through lentiviral infection. Error bars represent S.D. *, *p* < 0.05, **, *p* < 0.01

We applied survival analysis to the data on 71 hepatoma patients who received conservative surgery and then were monitored for up to 10 years. We observed a significant inverse correlation between HBP1 and AFP protein levels in hepatoma tissues by immunohistochemical staining (Fig. 1 a). As the degree of malignancy increased, the expression of HBP1 gradually decreased from phase I to phase IV under the TNM staging system. In contrast, AFP gradually increased from phase I to phase IV. We defined HBP1 or AFP expression greater than mean as HBP1 or AFP high expression group, and vice versa low expression group. Survival analysis demonstrated that HBP1 high expression, or AFP low expression was associated with higher survival rate (Fig. 1 b, top panel). The expression level of HBP1 decreased with the increase of tumor TNM stage, and the survival rate after surgery also decreased (Fig. 1 b, middle and bottom panels). In addition, HBP1 protein level in HBV positive (HBs Ag^+^) hepatoma patients was lower than that in HBV negative (HBs Ag^−^) hepatoma patients (Fig. 1 c). The data suggest that the inverse expressions of HBP1 and AFP correlate with relapse and survival in hepatoma patients. HBP1 may be an inhibitor in the development of HBV-associated hepatoma.

To determine whether HBP1 represses AFP expression, we overexpressed HBP1 in HepG2 and PLC5 cells (Fig. 1 d). AFP protein (in cytoplasm or supernatant) (left panel) and mRNA (right panel) levels were reduced by HBP1 overexpression in both HepG2 and PLC5 cells. To confirm the endogenous regulation of HBP1 on AFP expression, we knocked down the expression of HBP1 using short hairpin RNAs (shRNAs). As shown in Fig. 1 e, HBP1 knockdown resulted in increased levels of AFP protein (in cytoplasm or supernatant) (left panel) and mRNA (right panel) in the two cell lines. These results suggest that HBP1 inhibits AFP expression at the transcriptional level.

### HBP1 represses the AFP gene through binding an affinity site in the AFP promoter

**Fig. 2:**
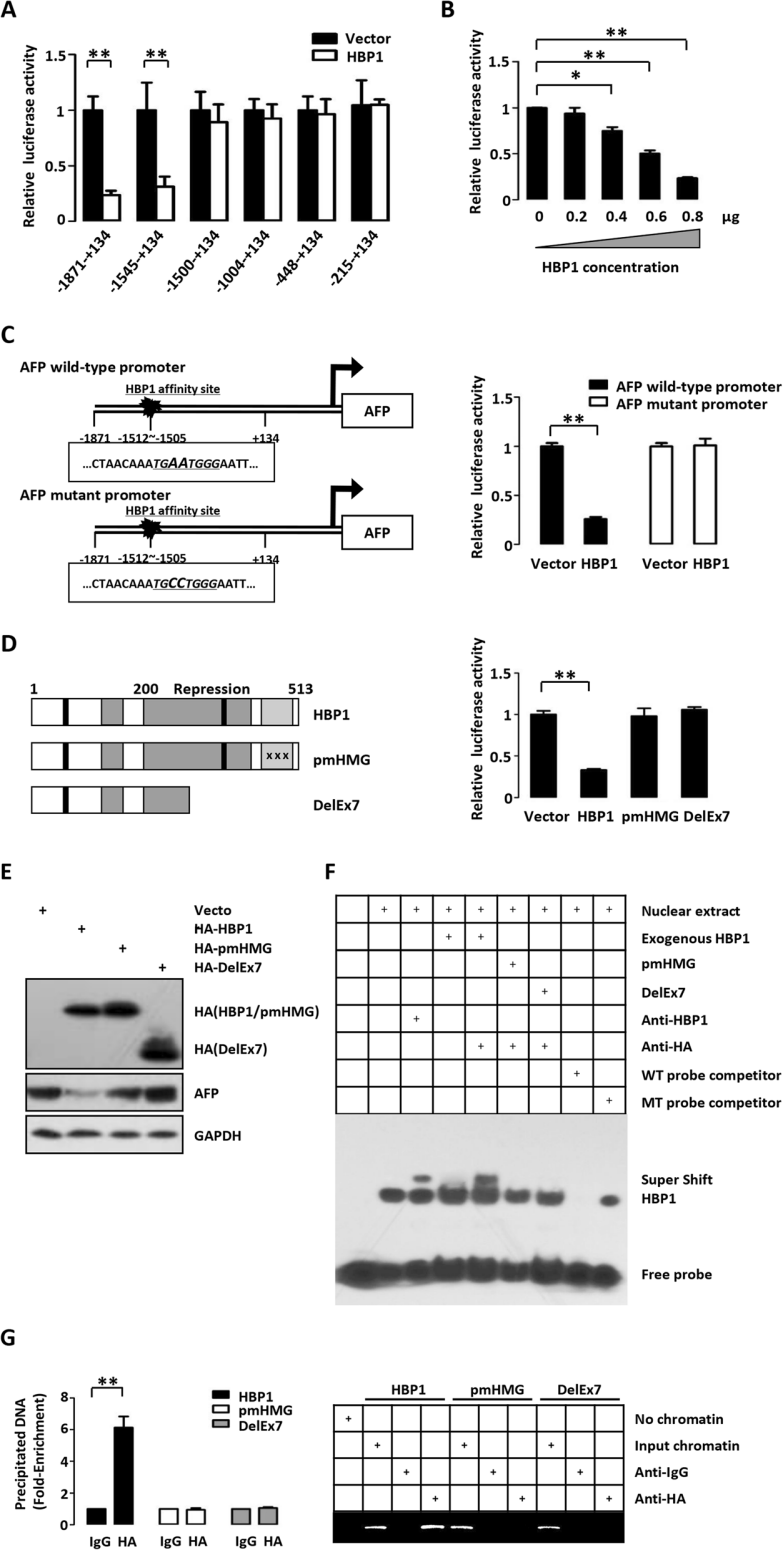
HBP1 represses the AFP gene through binding an affinity site in the AFP promoter. **a** Relative activity of HBP1 on the AFP promoters with various lengths. **b** HBP1 suppresses AFP promoter activity in a dose-dependent manner. Luciferase activity was detected in 293 T cells co-transfected with the indicated reporter genes and HBP1 plasmids. **c** The integrity of affinity site is indispensable for HBP1 suppressing AFP promoter in vivo. Shown is schematic diagram of the wild type AFP promoter and its mutant promoter (left panel) and the relative activities of HBP1 on the wild type AFP promoter and mutant AFP promoters (right panel). **d** Relative activities of HBP1 and associated mutants on the AFP promoter in co-transfected 293 T cells. Shown is schematic diagram of wild-type HBP1 and associated mutants (left panel). Luciferase activity was determined after transfection (right panel). **e** Expression of exogenous HBP1 decreases AFP protein level. Two hundred-ninety three T cells were transfected with HBP1 and associated mutants. The protein level was measured by Western blotting. **f** HBP1 occupies its affinity site in the AFP promoter. EMSA assays were performed by using a biotin-labeled double-stranded probe consisting of one HBP1 affinity site. Nuclear extracts from 293 T cells transfected with or without HBP1, pmHMG, or DelEx7 expression plasmid were used. Cold competitors were included in the indicated lanes at 200-fold excess. The presence of specific complexes, including supershifted HA or HBP1, was indicated. **g** HBP1 binding to the endogenous AFP promoter requires the HMG domain. ChIP assays were used to test the binding of exogenous HBP1 to endogenous AFP gene. Two hundred-ninety three T cells were transfected with HA-HBP1, HA-pmHMG or HA-DelEx7. The region from position − 1600 to position − 1448 contains the HBP1 affinity site and was analyzed by specific PCR and Realtime-PCR. Anti-HA antibody was used in the indicated lanes. Error bars represent S.D. *, *p* < 0.05, **, *p* < 0.01, ***, *p* < 0.001

We next investigated whether HBP1 regulates the promoter activity of AFP in a DNA-binding-dependent manner. We found that the AFP promoter contains an affinity site (TGAATGGG) for HBP1 at position − 1512 to − 1505 bp from the transcriptional start site. We co-transfected 293 T cells with plasmids expressing HBP1 and different deletion mutants of AFP promoter reporter genes (Fig. 2 a). HBP1 inhibited the reporter genes containing the HBP1 affinity site (− 1871 to + 134 and − 1545 to + 134), whereas HBP1 had no effect on the reporter genes lacking the HBP1 affinity site (− 1500 to + 134, − 1004 to + 134, − 448 to + 134 and − 215 to + 134). HBP1 also inhibited AFP promoter activity in a dose-dependent manner (Fig. 2 b).

To further verify the DNA binding requirement for the effects of HBP1, we constructed a mutant reporter for the AFP promoter with point mutations in the binding element at − 1510 (changing AA to CC). HBP1 inhibited the activity of the wild-type AFP promoter, but had no effect on the mutant (Fig. 2 c). To further investigate whether the transcriptional repression of HBP1 depends on DNA binding, we used two mutants of HBP1: pmHMG, which has three amino acid mutations in the HMG domain and lacks DNA binding ability, and DelEx7, which was isolated from breast cancer tissue and lacks the DNA binding domain and part of the repression domain [17, 20]. As shown in Fig. 2 d and e, wild-type HBP1 decreased the activity of AFP promoter and protein level, but overexpression of pmHMG and DelEx7 had no effect. Since HBP1 inhibited the activity of the AFP promoter, we tested whether HBP1 directly binds to the AFP promoter. Electrophoretic mobility shift assay (EMSA) (Fig. 2 f) and chromatin immunoprecipitation (ChIP) assay (Fig. 2 g) demonstrated that HBP1 bound directly to the specific affinity site in the AFP promoter in vivo and in vitro. In contrast, pmHMG and DelEx7 did not bind the AFP promoter.

### HBP1 acetylation at K419 is required for HBP1 suppression of AFP transcription

**Fig. 3:**
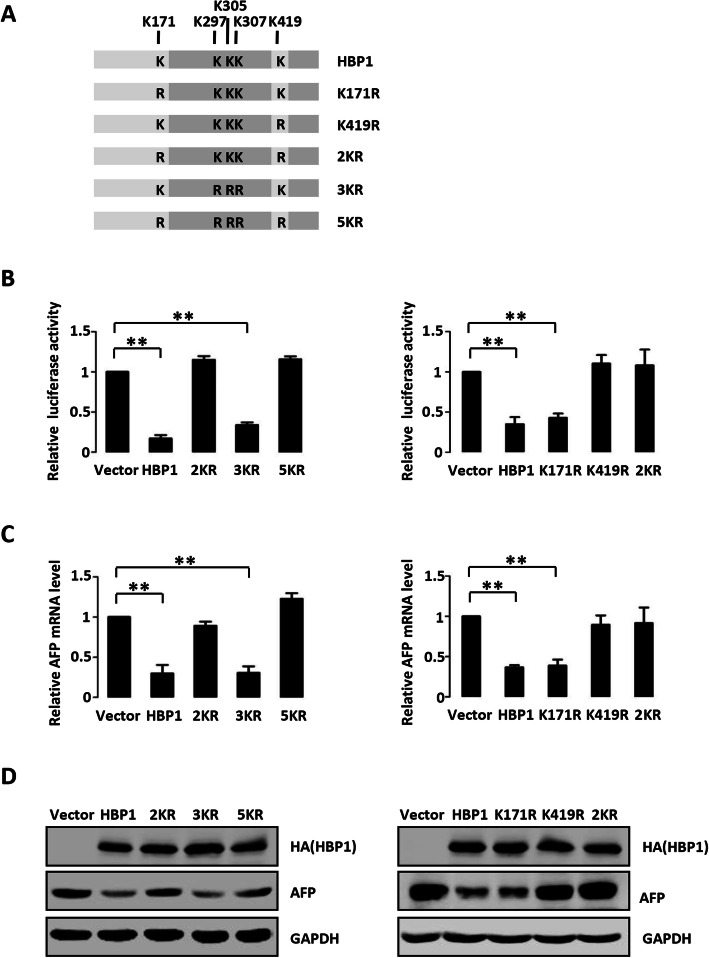
HBP1 acetylation at K419 is required for HBP1 suppression of AFP transcription. **a** Schematic diagram of wild-type HBP1 and associated point mutants. **b** The acetylation of HBP1 at K419 is indispensable for its suppression of AFP promoter. Two hundred-ninety three T cells were co-transfected with AFP promoter and HBP1 or mutant expression plasmids. Luciferase activity was determined after transfection. **c** The acetylation of HBP1 at K419 is indispensable for its suppression of AFP mRNA. The mRNA level of AFP was measured by Realtime-PCR in HepG2 cells transfected with HBP1 or associated mutant vectors individually. **d** The acetylation of HBP1 at K419 is indispensable for its suppression of AFP protein. HepG2 cells were transfected with HBP1 or associated mutant vectors. The protein levels of HBP1 and AFP were measured by Western blotting. Error bars represent S.D. **, *p* < 0.01

Our previous studies showed that HBP1 is acetylated by p300/CBP at five lysine sites (K292, K305, and K307 in the Repression domain, and K171 and K419 in the P domain). Acetylation of HBP1 is required for its transcriptional activity [26]. To investigate whether acetylation of HBP1 affects its transcriptional repression of AFP, we used various HBP1 mutants: K171R, K419R, 2KR (K171/419R), 3KR (K297/305/307R), and 5KR (K171/419/297/305/307R) (Fig. 3 a). We co-transfected the AFP luciferase reporter with either wild-type HBP1 or the HBP1 acetylation mutants into 293 T cells. Wild-type HBP1 repressed AFP promoter activity. The 3KR acetylation-deficient mutant in the Repression domain showed similar results as wild-type HBP1, whereas mutant 2KR and 5KR showed no repression of the AFP promoter-luciferase construct (Fig. 3 b, left panel). Consistent with these results, wild-type HBP1 and mutant 3KR decreased AFP mRNA and protein levels, however, overexpressing mutant 2KR or 5KR had no effect on AFP expression compared with control vector (Fig. 3 c and d, left panel). We further found that HBP1 acetylation at K419 was required for its suppression of AFP transcription (Fig. 3 b, right panel). Likewise, the regulation of AFP mRNA and protein levels (Fig. 3 c and d, right panel) depended on acetylation of HBP1 at K419. These data indicate that HBP1 acetylation at K419 is crucial for its repression of AFP transcription.

### HBx attenuates the suppression of HBP1 on AFP through inhibiting HBP1 binding to AFP promoter

**Fig. 4:**
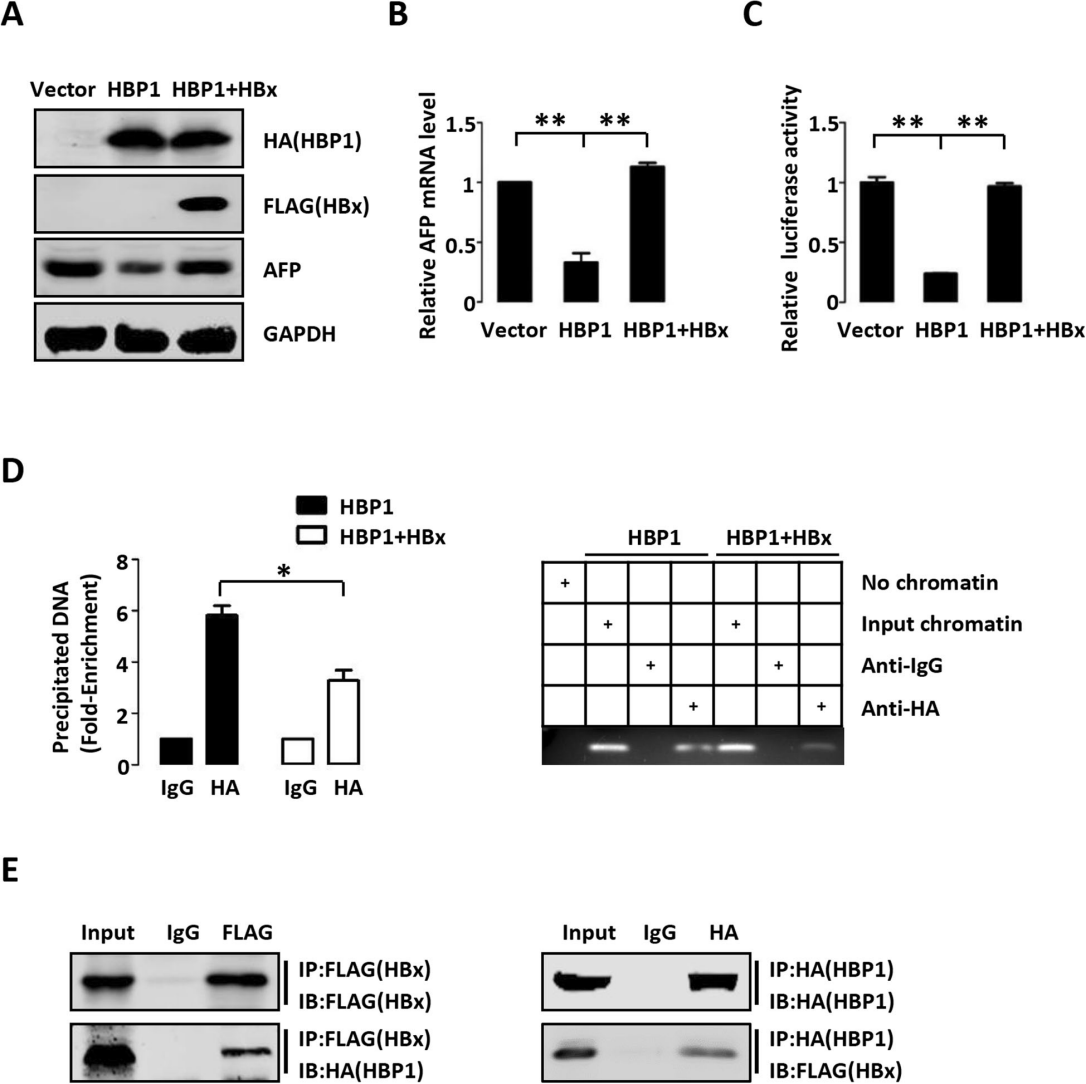
HBx attenuates the suppression of HBP1 on AFP through inhibiting binding of HBP1 to the AFP promoter. **a** HBx rescues the suppression of HBP1 on AFP protein. HepG2 cells were co-transfected HBP1 with or without HBx expression plasmid. The protein levels of HBP1, AFP and HBx were measured by Western blotting. **b** HBx rescues the suppression of HBP1 on AFP mRNA. The mRNA level of AFP was measured by Realtime-PCR in HepG2 cells transfected with HBP1 with or without HBx expression plasmid. **c** HBx rescues the suppression of HBP1 on AFP promoter. Two hundred-ninety three T cells were co-transfected with AFP promoter and HBP1 with or without HBx expression plasmid. Luciferase activity was determined after transfection. **d** HBx inhibits the interaction between HBP1 and AFP promoter. Two hundred-ninety three T cells were transfected with HA-HBP1 with or without HBx expression plasmid. The region from position − 1600 to position − 1448 contains the HBP1 affinity site and was analyzed by specific PCR. **e** HBP1 interacts with HBx. Two hundred-ninety three T cells were co-transfected with HA-HBP1 and FLAG-HBx. IP assay was carried out by using anti-FLAG/HA antibody and followed by Western blotting with anti-HA/FLAG antibody. The same samples were immunoblotted against FLAG/HA to determine immunoprecipitation efficiency. Error bars represent S.D. *, *p* < 0.05, **, *p* < 0.01

The development of hepatoma is closely related to hepatitis B virus (HBV) infection [29]. HBx is a HBV regulatory protein that up-regulates AFP expression and promotes the development of HBV-associated hepatoma [30, 31]. To determine whether HBx promotes the development of hepatoma through inhibiting the downregulation of AFP by HBP1, we overexpressed HBP1 with or without HBx in HepG2 cells. As shown in Fig. 4 a–4c, HBx rescued the HBP1-mediated decrease of AFP protein level, mRNA level and promoter activity. We next examined whether HBx affects the binding of HBP1 to the AFP promoter. ChIP results confirmed that HBx reduced the binding of HBP1 to the AFP promoter (Fig. 4 d). We also performed co-IP assays and found that exogenous HBx could interact with HBP1 (Fig. 4 e). Therefore, we speculated that HBx may inhibit the binding of HBP1 to the AFP promoter by interacting with HBP1. Together with Fig. 1 c, HBP1 protein level in HBs Ag^+^ hepatoma patients was lower than that in HBs Ag^−^ hepatoma patients, indicating that the inhibition of AFP by HBP1 might reduce in HBs Ag^+^ hepatoma patients because of low HBP1, thereby promoting hepatoma progression.

### Icaritin promotes the suppression of HBP1 on AFP through enhancing HBP1 binding to AFP promoter

**Fig. 5:**
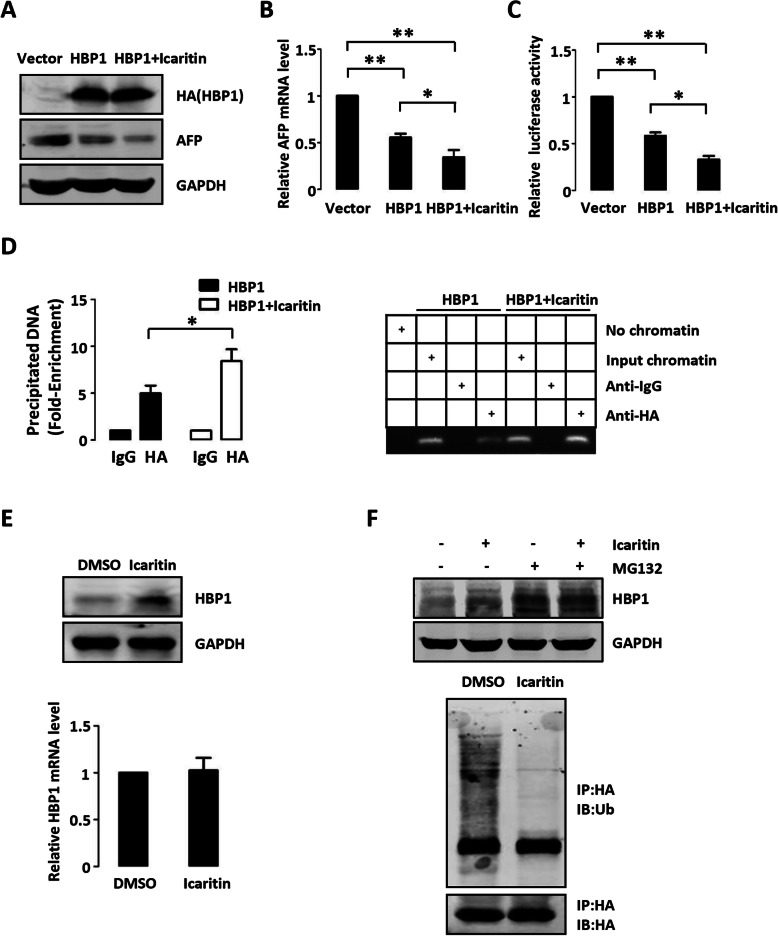
Icaritin promotes the suppression of HBP1 on AFP through enhancing HBP1 binding to AFP promoter. **a** Icaritin promotes the suppression of HBP1 on AFP protein. HepG2 cells were transfected HBP1 with or without Icaritin treatment. The protein levels of HBP1 and AFP were measured by Western blotting. **b** Icaritin promotes the suppression of HBP1 on AFP mRNA. The mRNA level of AFP was measured by Realtime-PCR in HepG2 cells transfected HBP1 with or without Icaritin treatment. **c** Icaritin promotes the suppression of HBP1 on AFP promoter. Two hundred-ninety three T cells were co-transfected with AFP promoter and HBP1 with or without Icaritin treatment. Luciferase activity was determined after transfection. **d** Icaritin promotes the interaction between HBP1 and AFP promoter. Two hundred-ninety three T cells were transfected HA-HBP1 with or without Icaritin treatment. The region from position − 1600 to position − 1448 contains the HBP1 affinity site and was analyzed by specific PCR. **e** HBP1 protein level is elevated in the presence of Icaritin. HepG2 cells were treated with Icaritin. The protein level of HBP1 was measured by Western blotting (top panel). The mRNA level of HBP1 was measured by Realtime-PCR (bottom panel). **f** Icaritin inhibits HBP1 ubiquitination-mediated proteasome degradation. HepG2 cells were treated with Icaritin with or without MG132, the protein level of HBP1 was measured by Western blotting (top panel). Two hundred-ninety three T cells were co-transfected HA-HBP1, FLAG-MDM2 with or without Icaritin treatment for 24 h and then exposed to MG132 for another 6 h prior to lysis. HBP1 protein was then isolated by immunoprecipitation and analyzed by anti-Ub antibody (bottom panel). Error bars represent S.D. *, *p* < 0.05, **, *p* < 0.01

Icaritin is an active ingredient of the Chinese herb epimedium that has been proven to have a wide range of biological and pharmacological functions, such as enhancing immunity and antioxidative and anticancer functions [32]. Previous studies reported that Icaritin inhibits the growth of liver cancer cells through promoting the apoptosis of hepatoma cells by activating the caspase pathway and inhibiting the IL-6/Jak2/Stat3 signaling pathway [33, 34]. Zhang et al. demonstrated that Icaritin reduces the expression of AFP by promoting miRNA-mediated degradation of AFP mRNA [35]. We thus next examined whether Icaritin interferes with the suppression of HBP1 on AFP. We overexpressed HBP1 with or without Icaritin treatment in HepG2 cells and examined the protein level, mRNA level and promoter activity of AFP. As shown in Fig. 5 a–5c, HBP1 more significantly inhibited AFP expression in the presence of Icaritin compared with HBP1 alone. ChIP assay showed that Icaritin enhanced the binding of HBP1 to the AFP promoter (Fig. 5 d). To further investigate how Icaritin enhances the transcriptional repression of HBP1, we evaluated the protein and mRNA levels of HBP1 in HepG2 cells after Icaritin treatment. As shown in Fig. 5 e, Icaritin increased HBP1 protein expression but had no effect on mRNA level. Furthermore, Icaritin inhibited HBP1 ubiquitination-mediated proteasome degradation (Fig. 5 f). Thus, we concluded that Icaritin increases HBP1 expression by inhibiting the ubiquitination of HBP1, thereby enhancing HBP1 binding to AFP promoter.

### The repression of AFP by HBP1 attenuates AFP effect on PTEN, MMP9 and caspase-3 protein levels in hepatoma cells

**Fig. 6:**
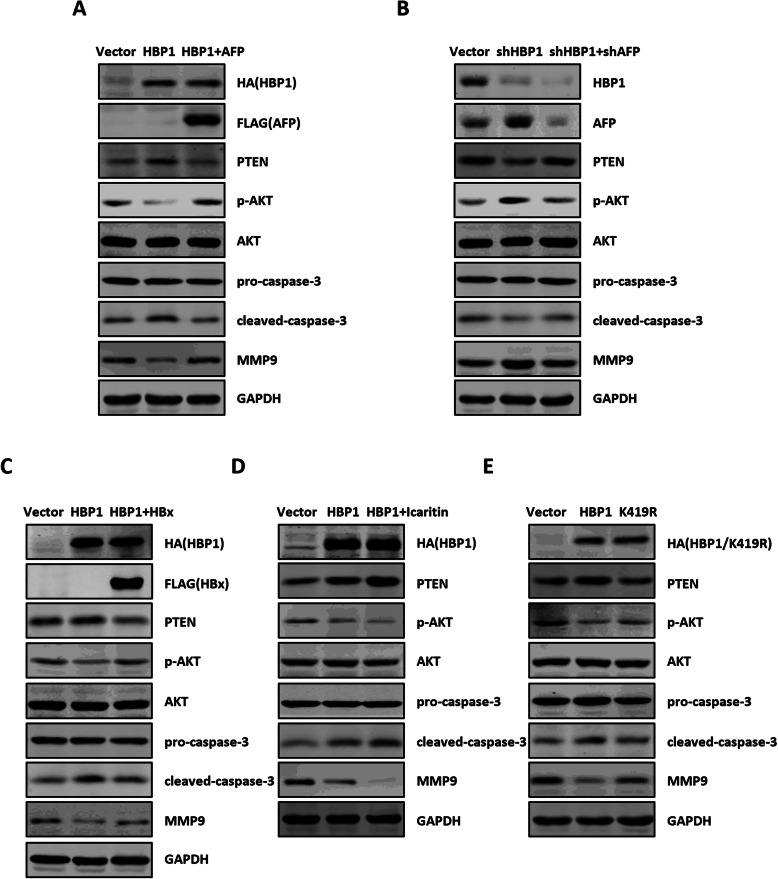
HBP1 inhibits the functions of PTEN and caspase-3 through suppressing AFP in hepatoma cells. **a** AFP rescues the effect of HBP1 on PTEN, caspase-3, and MMP9 protein levels. HepG2 cells were co-transfected HBP1 with or without AFP expression plasmid. The protein levels of HBP1, AFP, PTEN, phospho-AKT, AKT, pro-caspase-3, cleaved-caspase-3 and MMP9 were measured by Western blotting. **b** AFPshRNA rescues the effect of HBP1shRNA on PTEN, caspase-3, and MMP9 protein levels. HepG2 cells were co-transfected HBP1shRNA with or without AFPshRNA expression plasmid. The protein levels of HBP1, AFP, PTEN, phospho-AKT, AKT, pro-caspase-3, cleaved-caspase-3 and MMP9 were measured by Western blotting. **c** HBx rescues the effect of HBP1 on PTEN, caspase-3, and MMP9 protein levels. HepG2 cells were co-transfected HBP1 with or without HBx expression plasmid. The protein levels of HBP1, AFP, PTEN, phospho-AKT, AKT, pro-caspase-3, cleaved-caspase-3 and MMP9 were measured by Western blotting. **d** Icaritin enhances the effect of HBP1 on PTEN, caspase-3, and MMP9 protein levels. HepG2 cells transfected with HBP1 were treated with or without Icaritin. The protein levels of HBP1, AFP, PTEN, phospho-AKT, AKT, pro-caspase-3, cleaved-caspase-3 and MMP9 were measured by Western blotting. **e** HBP1 acetylation at K419 is required for the effect of HBP1 on PTEN, caspase-3, and MMP9 protein levels. HepG2 cells were transfected HBP1 or K419R expression plasmid. The protein levels of HBP1, AFP, PTEN, phospho-AKT, AKT, pro-caspase-3, cleaved-caspase-3 and MMP9 were measured by Western blotting

Previous studies reported that AFP exerts its tumor promotion through interacting with PTEN, MMP9, and caspase-3 and blocking their functions on the PI3K/AKT, metastasis and caspase signaling [8, 9]. We then tested whether the repression of AFP by HBP1 attenuates AFP effect on PTEN, MMP9 and caspase-3 in HepG2 cells. As shown in Fig. 6 a, HBP1 overexpression increased PTEN protein expression and decreased phosphorylated AKT level. Meanwhile, HBP1 enhanced pro-caspase-3 processing to cleaved-caspase-3 and decreased the protein level of MMP9, whereas co-expressing AFP rescued the HBP1-mediated changes in PTEN, caspase-3, and MMP9. Furthermore, knockdown of HBP1 increased AFP expression, thereby decreased PTEN and cleaved-caspase-3 levels, and increased phosphorylated AKT and MMP9 levels, but there was no effect when AFP was also knocked down (Fig. 6 b). We also examined the K419R mutant and found that it had no effect on expression of the proteins (Fig. 6 e). These results indicated that HBP1 increases protein levels of PTEN and cleaved-caspase-3 and decreases protein level of MMP9 through suppressing AFP, and HBP1 exerts the role depending on its acetylation at K419.

We also tested the role of HBx or Icaritin in regulating HBP1-mediated expression of these proteins. As shown in Fig. 6 c and d, HBx rescued HBP1-mediated expression of these proteins, while Icaritin enhanced HBP1-mediated expression of these proteins, suggesting that HBx or Icaritin influences PTEN/AKT, caspase-3, and MMP9 signals through regulating HBP1-AFP axis.

### HBP1 inhibits malignancy through suppressing AFP in hepatoma cells

**Fig. 7:**
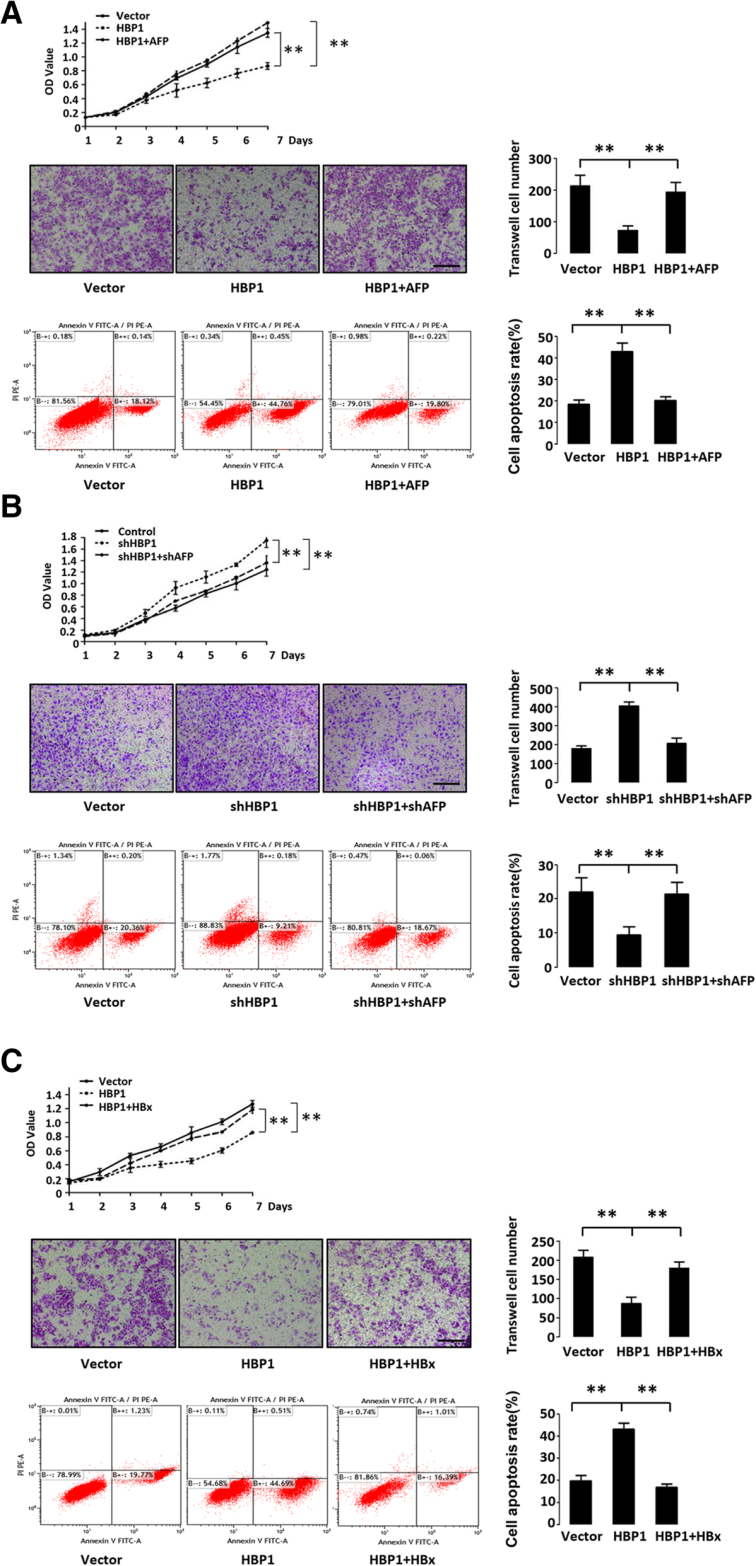

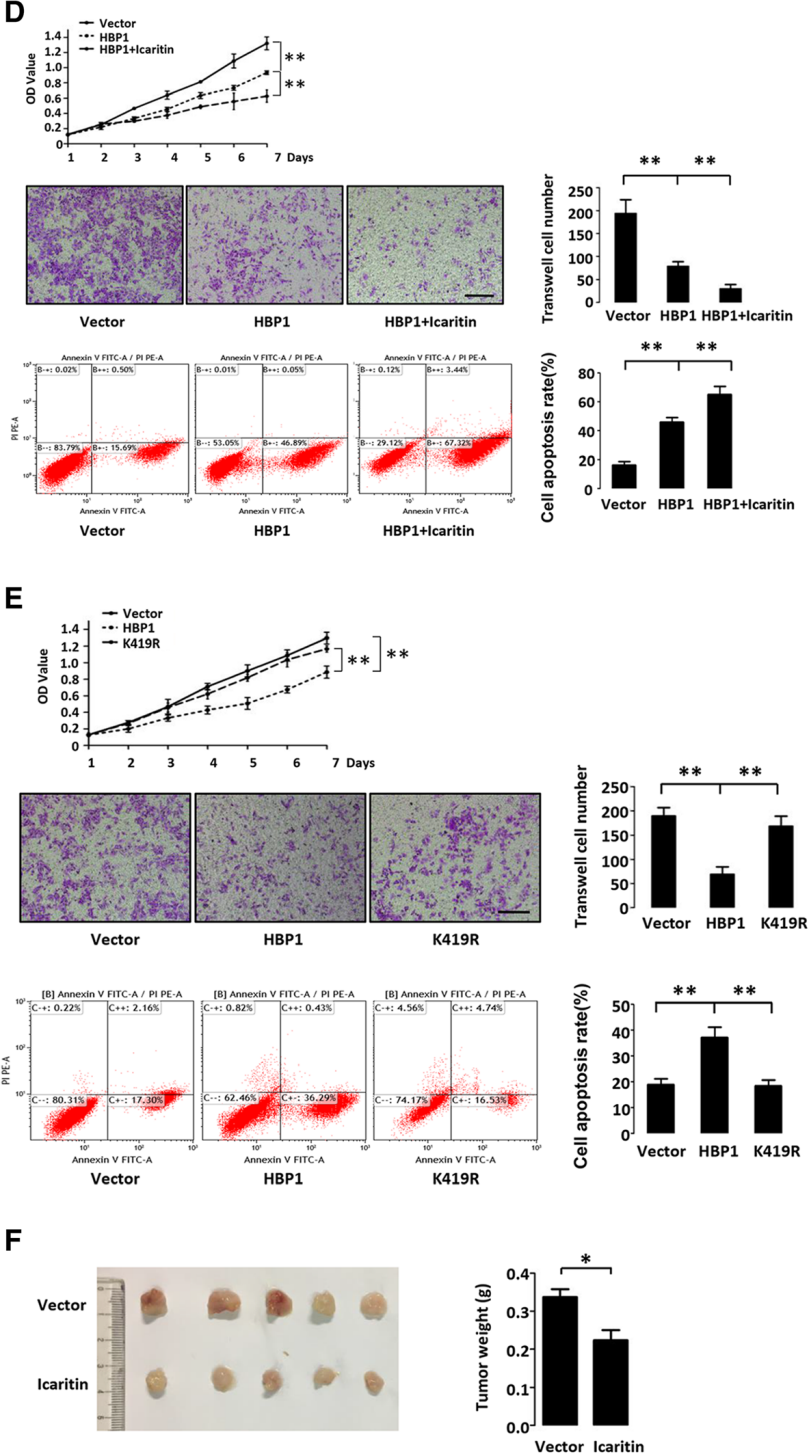
(See Fig. 7 continuation on next page.)

Since HBP1 upregulates AKT and caspase-3 signals and decreases metastasis-related protein of MMP9 through suppressing AFP, we next investigated the role of HBP1 suppression on AFP expression in cell viability, migration and apoptosis in HepG2 cells. Consistent with previous studies, HBP1 decreased cell viability and cell migration as shown by growth curve (Fig. 7 a, top panel) and Transwell assay (Fig. 7 a, middle panel), also increased cell apoptosis response to H_2_O_2_ induction, as demonstrated by FACS (Fig. 7 a, bottom panel). AFP rescued the HBP1-induced decrease of cell proliferation and migration, and also rescued the increase of cell apoptosis (Fig. 7 a). HBP1-knockdown cells showed higher growth rate and cell migration ability, but lower apoptosis rate response to H_2_O_2_ induction, while AFP knockdown by shRNA in HBP1-knockdown cells rescued the elevated proliferation and migration, and the decreased apoptosis induced by knockdown of HBP1 (Fig. 7 b).

To further determine whether HBx or Icaritin treatment alters the degree of malignancy in hepatoma cells, HepG2 cells were stably transfected with HBP1 with or without lentivirus expressing HBx or Icaritin treatment. As shown in Fig. 7 c and d, HBx rescued HBP1-mediated decrease of cell proliferation and migration, and the increase of cell apoptosis, while Icaritin enhanced HBP1-mediated decrease of cell proliferation and migration, and the increase of cell apoptosis. In addition, Icaritin inhibited tumorigenesis of HepG2 cells in the nude mice (Fig. 7 f). These results indicated that HBP1 inhibits malignancy in hepatoma cells through suppressing AFP, and HBx or Icaritin influences hepatoma cell malignancy through regulating HBP1-AFP axis. We also examined the K419R mutant and confirmed that the effect of HBP1 on cell proliferation, migration and apoptosis is dependent on its acetylation of K419 (Fig. 7 e).

5-fluorouracil (5-FU) is an anti-liver cancer drugs widely used in clinic. In order to test if 5-FU displays similar effects of Icaritin on HBP1 or AFP, we performed Western blotting, Realtime PCR, ChIP, and MTT assays with HBP1-overexpressing HepG2 cells treated with or without 5-FU. As shown in Fig.S1A-1F, 5-FU also increased HBP1 protein expression by inhibiting HBP1 ubiquitination-mediated proteasome degradation, thereby enhances HBP1 repression on AFP promoter. Furthermore, 5-FU enhanced HBP1-mediated expression of PTEN/AKT, caspase-3, and MMP9 proteins (Fig.S1G), and enhanced HBP1-mediated decrease of cell proliferation (Fig.S1H). These results indicated that 5-FU, which displays similar effects of Icaritin on HBP1 or AFP, may inhibit hepatoma progression though regulating HBP1-AFP axis.

### HBP1 deletion aggravates DEN/CCl_4_-induced hepatoma

To further investigate the function of HBP1 in the development of hepatoma, we constructed HBP1 knockout mice using the CRISPR/Cas9 system. The removal of 194 base pairs in the third exon of the HBP1 gene prevented the HBP1 gene transcription, resulting in the deletion of HBP1 protein. After genetic identification of newborn mice, we selected two groups of wild-type and HBP1-deficient mice and randomly assigned six wild-type and six HBP1-deficient mice per group. One group was treated with DEN/CCl_4_ to construct a mouse hepatic fibrosis and hepatoma models, and the other group was treated with saline/olive oil as a negative control. About 12 weeks later, the mouse hepatic fibrosis model was firstly established. We sacrificed the mice and excised the livers for analyses. As shown in Fig. S2A, more inflammatory cell infiltrations and necrotic foci were formed in the liver of HBP1-deficient mice treated with DEN/CCl_4_ compared with that of HBP1 wild-type mice. Masson staining and Sirius Red staining showed that hepatic fibrosis only occurred in the mouse group treated with DEN/CCl_4_, and more severe hepatic fibrosis occurred in HBP1-deficient mice (Fig. S2B). There was no liver damage and fibrosis in wild-type or HBP1-deficient mice in the groups not treated with DEN/CCl_4_ (Fig. S3A, S3B). We then detected the protein and mRNA levels of several inflammatory factors associated with hepatic fibrosis in the liver of mice. As shown in Fig. S2C and S2D, the protein and mRNA levels of TypeIcollagen, Type III collagen and TNF-α were increased in the liver of HBP1-deficient mice, while IL-1β expression was unchanged compared with levels in wild-type HBP1 mice. We also tested ALT and AST levels in mice serum. ALT and AST levels increased after DEN/CCl_4_ treatment and both levels increased more significantly in HBP1-deficient mice compared with wild-type mice (Fig. S2E), indicating that HBP1-deficient mice had more severe liver function damage after DEN/CCl_4_ treatment, whereas ALT and AST levels did not increase in the group without DEN/CCl_4_ treatment (Fig. S3C).

**Fig. 8:**
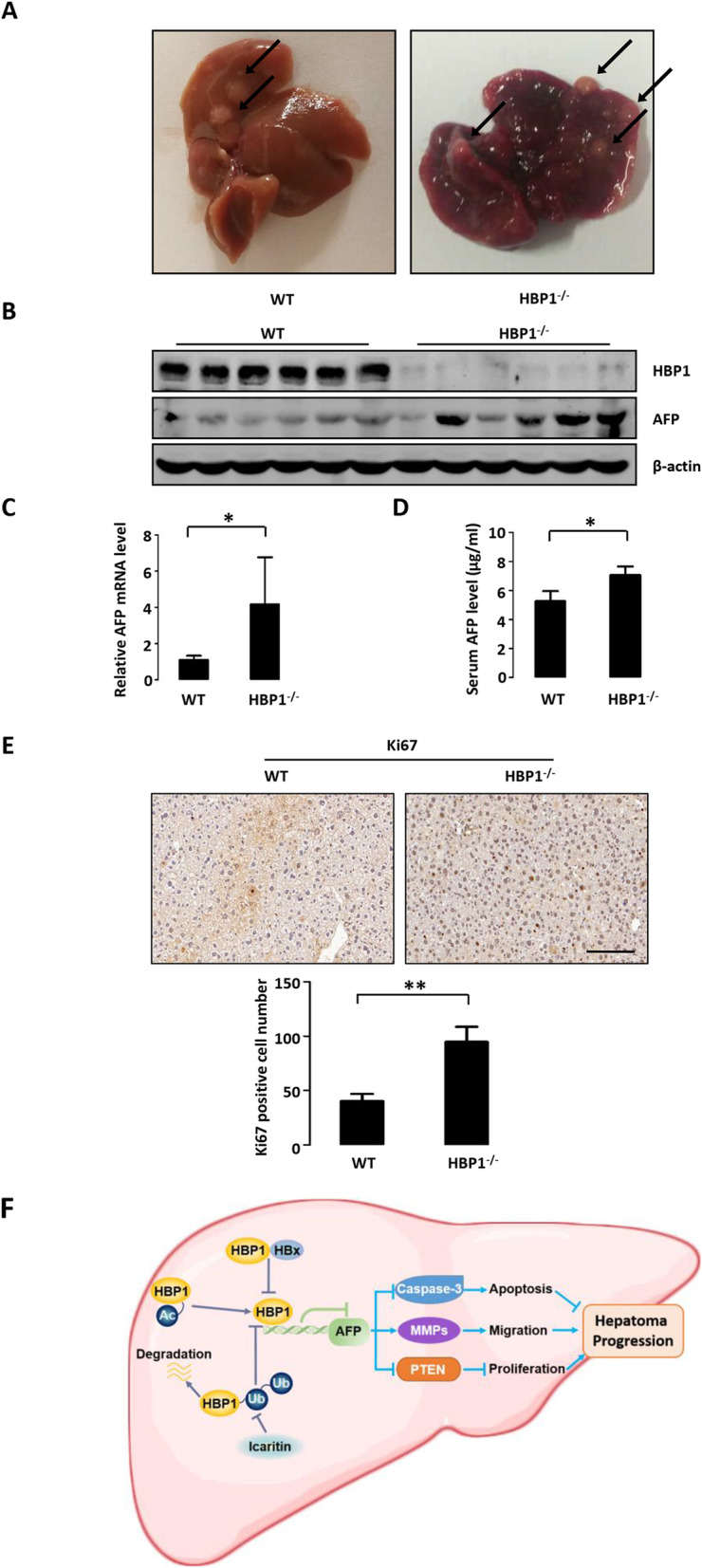
HBP1 deletion aggravates DEN/CCl_4_-induced hepatoma. **a** Representative images of hepatoma sections from 20 weeks after DEN/CCl_4_ treatment in wild type or HBP1^−/−^ mice. **b** The protein levels of HBP1 and AFP in the hepatoma described in Fig. 8**a** were measured by Western blotting. β-actin was used as a loading control. **c** The mRNA level of AFP was measured by Realtime-PCR in the hepatoma described in Fig. 8**a**. **d** Serum AFP level in the mice described in Fig. 8**a**. **e** The IHC analyses of Ki67 in the mice described in Fig. 8**a**. **f** Model of the role of HBP1 in inhibiting the occurrence and development of hepatoma by repressing the expression of AFP. Scale bar, 100 μm. Bar graphs show numbers of Ki67-positive cells per 100 × field. Error bars represent S.D. *, *p* < 0.05, **, *p* < 0.01

In separate experiments, mice were treated with DEN/CCl_4_ or saline/olive oil (as a negative control) for longer (about 20 weeks) to induce hepatoma. HBP1-deficient mice had more liver tumor tissue blocks, and the tumor tissue volume was larger compared with wild-type mice (Fig. 8 a). In addition, multiple nodules were clearly visible on the HBP1-deficient mouse liver surface, while the wild-type mouse liver was relatively smooth. As shown in Fig. 8 b and c, the protein level and mRNA level of AFP were increased in the liver of HBP1-deficient mice. Since AFP is a serum marker of primary liver cancer, we evaluated the AFP content in mouse serum using ELISA and found that serum AFP level of HBP1-deficient mice was higher than that of wild-type mice (Fig. 8 d). IHC staining of paraffin sections showed that Ki67 expression was higher in HBP1-deficient mice compared with wild-type mice (Fig. 8 e), indicating that HBP1-deficient mouse hepatoma was more malignant in the DEN/CCl_4_-induced hepatoma model. Together, the data suggest that HBP1 deletion aggravates DEN/CCl_4_-induced liver damage, hepatic fibrosis and hepatoma in mice. Our results are thus consistent with a model in which HBP1 inhibits hepatoma by repressing the expression of AFP (see model in Fig. 8 f).

## Discussion

It has been reported that HBP1 maintains a proliferation barrier through regulating G1 progression in differentiated liver tissue, but the mechanism is unclear [36]. Our current study expands the possibilities for the negative regulation of proliferation by HBP1 through its inhibition of AFP expression. Here, we show that HBP1 regulates AFP expression at the transcriptional level and inhibits the development of hepatoma. The inverse relationship of HBP1 and AFP in hepatoma patients correlates with hepatoma relapse. HBP1 low expression and/or AFP high expression in hepatoma can be correlated with low survival rate. Our experimental data demonstrate that AFP is a target gene of the transcription factor HBP1. HBP1 directly binds to the promoter of AFP to inhibit AFP gene expression. Using multiple point mutants of HBP1, we found that transcriptional inhibition of AFP by HBP1 is dependent on HBP1 acetylation at K419 (Figs. 1, 2, 3). In addition, HBx, a HBV regulatory protein that promotes the development of HBV-associated hepatoma, can interact with HBP1, inhibiting the binding of HBP1 to the AFP promoter region and leading to up-regulated AFP expression. Icaritin, an active ingredient of the Chinese herb epimedium, inhibits the ubiquitination of HBP1 and enhances HBP1 protein stability, resulting in downregulated protein level of AFP. Together, HBx or Icaritin mediates proliferation and migration of hepatoma cells through regulating the HBP1-AFP axis (Figs. 4-5). The repression of AFP by HBP1 attenuates AFP effect on PTEN, MMP9 and caspase-3, thus inhibits proliferation and migration, and induces apoptosis response to oxidative stress in hepatoma cells (Figs. 6-7). We constructed HBP1-deficient mice to investigate the role of HBP1 in the development of hepatoma. We found that AFP protein level increased in serum and liver in HBP1-deficient mice after DEN/CCl_4_ induction. Furthermore, HBP1-deficient mice with DEN/CCl_4_ induction developed more severe liver damage, fibrosis, and tumorigenesis (Fig. 8). Thus, the variation in the expression levels of HBP1 and AFP, which are both associated with cell proliferation, could have a lasting impact on tumorigenic progression.

In previous studies, HBP1 was described as a tumor suppressor that is activated by p38-MAPK and Pim-1, leading to premature cell decay and apoptosis [24, 25]. HBP1 expression is inhibited in a variety of human malignancies, and HBP1 gene mutation is found in breast cancer, suggesting that the mutation or loss of HBP1 may be one of the causes of cell transformation. In the present study, we found that HBP1 expression was also downregulated in hepatoma tissues, and overexpression of HBP1 in hepatoma cells transcriptionally inhibited AFP expression and decreased cell proliferation and migration. In the process of establishing a mouse hepatoma model, we found that the expression of HBP1 was gradually reduced, while AFP expression increased gradually (Fig. S4), suggesting that the function of HBP1 is gradually suppressed in the process of hepatitis-cirrhosis-hepatoma, and the deregulation of the HBP1-AFP axis contributes to hepatoma progression.

Chronic hepatitis caused by HBV infection is considered to be the main cause of hepatoma. HBV can activate the oncogenic pathway by integrating DNA into the genome of the host cell and causing gene mutations. Previous studies have reported that HBx can interact with the tumor suppressor p53, block p53 nuclear translocation, and abolish the transcriptional repression of p53 on AFP [36–39]. In this study, we used HBx to mimic the effects of HBV infection. We demonstrated that HBx directly binds to HBP1, inhibits the binding of HBP1 to the AFP gene promoter and up-regulates AFP expression. Our study highlights a novel mechanism underlying HBx regulatory effects in hepatoma. In addition, previous studies showed HBx can inhibit apoptosis and promote tumorigenesis through other mechanisms [40, 41]. Therefore, we believe that due to the presence of HBx, treating HBV with drugs that only target HBV replication cannot completely eliminate the effects of HBV in promoting hepatoma. Alternate strategies should be pursued to inhibit the protein function of HBx, thereby reducing the possibility of hepatitis B patients developing hepatoma.

In this study, DEN/CCl_4_ was used to induce a mouse hepatoma model. DEN is a strong carcinogen, and long-term stimulation with CCl_4_ causes acute/chronic hepatitis in mice. Studies have shown that 70–90% of patients with hepatoma show symptoms of cirrhosis, and more than 10% of patients with cirrhosis eventually develop hepatoma [42–44]. Our study found that HBP1-deficient mice developed more severe liver fibrosis. This indicates that HBP1 has a certain protective effect on the normal function of the liver in the hepatitis caused by DEN/CCl_4_ treatment. Deletion of HBP1 is more likely to cause collagen (Type I and Type III) secretion and deposition, leading to the occurrence of cirrhosis in mice. However, under normal growth conditions, HBP1-deficient mice did not show abnormalities compared with wild-type mice (Fig. S3). This suggests that HBP1 may not affect the growth and development of the liver, but instead HBP1 has a protective effect on the liver when liver cells are exposed to external stimuli such as inflammatory factors and carcinogens. Our results indicate that the presence of HBP1 protects the liver and delays the progression of hepatitis to cirrhosis, finally inhibiting tumorigenesis.

We also found that high expression of HBP1 correlated with increased survival and prognosis in the hepatoma patient group examined in this study. Our results demonstrate that Icaritin enhances the stability of HBP1 and up-regulates the protein level of HBP1, thereby exerting anticancer activity.

## Conclusions

The deregulation of AFP by HBP1 is associated with hepatoma cell metastasis and poor survival of hepatoma patients. Under normal conditions, HBP1 inhibits tumorigenesis by repressing AFP expression. During hepatoma progression, this biological process could be reversed by aberrantly low HBP1 expression, which promotes AFP effect on PTEN, MMP9 and caspase-3, thus induces proliferation and migration, and inhibits apoptosis in hepatoma cells. The identification of a mechanism by which high HBP1 expression inhibits hepatoma progression opens up new therapeutic avenues for hepatoma therapy. We suggest that HBP1 expression level may be a useful indicator in the treatment and prognosis monitoring of hepatoma to better improve the prognosis of patients with hepatoma.

## Supplementary Information

**Additional file 1: Figure S1.** 5-FU promotes the suppression of HBP1 on AFP through enhancing HBP1 binding to AFP promoter. **A-C**. 5-FU promotes the suppression of HBP1 on AFP protein (A), mRNA (B) and promoter (C). HepG2 cells were transfected HBP1 with or without 5-FU treatment. The protein levels of HBP1 and AFP were measured by Western blotting. The mRNA level of AFP was measured by Realtime-PCR. The luciferase activities were detected in 293 T cells co-transfected with AFP promoter and HBP1 with or without 5-FU treatment and expressed as the means ± S.D. of the means from three experiments. (D) 5-FU promotes the interaction between HBP1 and AFP promoter. Two hundred ninety-three T cells were transfected HA-HBP1 with or without 5-FU treatment. The region from position − 1600 to position − 1448 contains the HBP1 affinity site and was analyzed by specific PCR. (E) HBP1 protein level is elevated in the presence of 5-FU. HepG2 cells were treated with different concentrations of 5-FU. The protein level was measured by Western blotting (top panel). The mRNA level was measured by Realtime-PCR (bottom panel). (F) 5-FU inhibits HBP1 ubiquitination-mediated proteasome degradation. HepG2 cells were treated with 5-FU with or without MG132. The protein level was measured by Western blotting (top panel). Two hundred ninety-three T cells were transfected HA-HBP1 with or without 5-FU treatment for 24 h and then exposed to MG132 for another 6 h prior to lysis. HBP1 protein was isolated by immunoprecipitation and analyzed by anti-Ub antibody (bottom panel). (G) 5-FU enhances the effect of HBP1 on PTEN, caspase-3, and MMP9 protein levels. HepG2 cells transfected with HBP1 were treated with or without 5-FU. The protein levels were measured by Western blotting. (H) 5-FU enhances HBP1-mediated decrease of cell proliferation. MTT assay was conducted with HepG2 cells stably transfected with control vector, HBP1 with or without 5-FU treatment. Error bars represent S.D. *, *p* < 0.05, **, *p* < 0.01.

**Additional file 2: Figure S2.** HBP1 deletion aggravates DEN/CCl_4_-induced hepatic fibrosis. (A) Representative H.E. staining of liver sections from 12 weeks after DEN/CCl_4_ treatment in wild type or HBP1^−/−^ mice. Scale bar, 100 μm. (B) Representative images of Masson’s trichrome staining and Sirius red staining of liver sections from the mice described in **Figure S2A.**. Scale bar, 200 μm. (C) The protein levels of HBP1, TypeIcollagen, Type III collagen, IL-1 and TNF-α in the mice liver described in **Figure S2A.** were measured by Western blotting. β-actin was used as a loading control. (D) The mRNA levels of TypeIcollagen, Type III collagen, IL-1 and TNF-α were measured by Realtime-PCR in the mice liver described in **Figure S2A**. (E) Serum ALT and AST levels of the mice described in **Figure S2A**. The means ± S.D. are shown (*n* = 5). Error bars represent S.D. *, *p* < 0.05, **, *p* < 0.01.

**Additional file 3: Figure S3.** HBP1 deletion has no effect on liver morphology and function in mice without DEN/CCl_4_ treatment. (A) Representative H.E. staining of liver sections from 12 weeks after saline treatment in wild type or HBP1^−/−^ mice. Scale bar, 100 μm. (B) Representative images of Masson’s trichrome staining and Sirius red staining of liver sections from the mice described in Figure S3A. Scale bar, 200 μm. (C) Serum ALT and AST levels of the mice described in Figure S3A. The means ± S.D. are shown (*n* = 5). Error bars represent S.D.

**Additional file 4: Figure S4.** There is a negative correlation between the expression of HBP1 and AFP in DEN/CCl_4_-induced hepatoma in mice. The protein levels of HBP1 and AFP in the liver were measured by Western blotting during DEN/CCl_4_ induction. β-actin was used as a loading control.

## Data Availability

Data sharing is not applicable to this article as no datasets were generated or analyzed during the current study.
