# Supplementary figures and images for: HBP1-mediated transcriptional repression of AFP inhibits hepatoma progression
Source: J Exp Clin Cancer Res. 2021 Apr 1;40:118. doi: 10.1186/s13046-021-01881-2 (PMC8015059; doi:10.1186/s13046-021-01881-2)

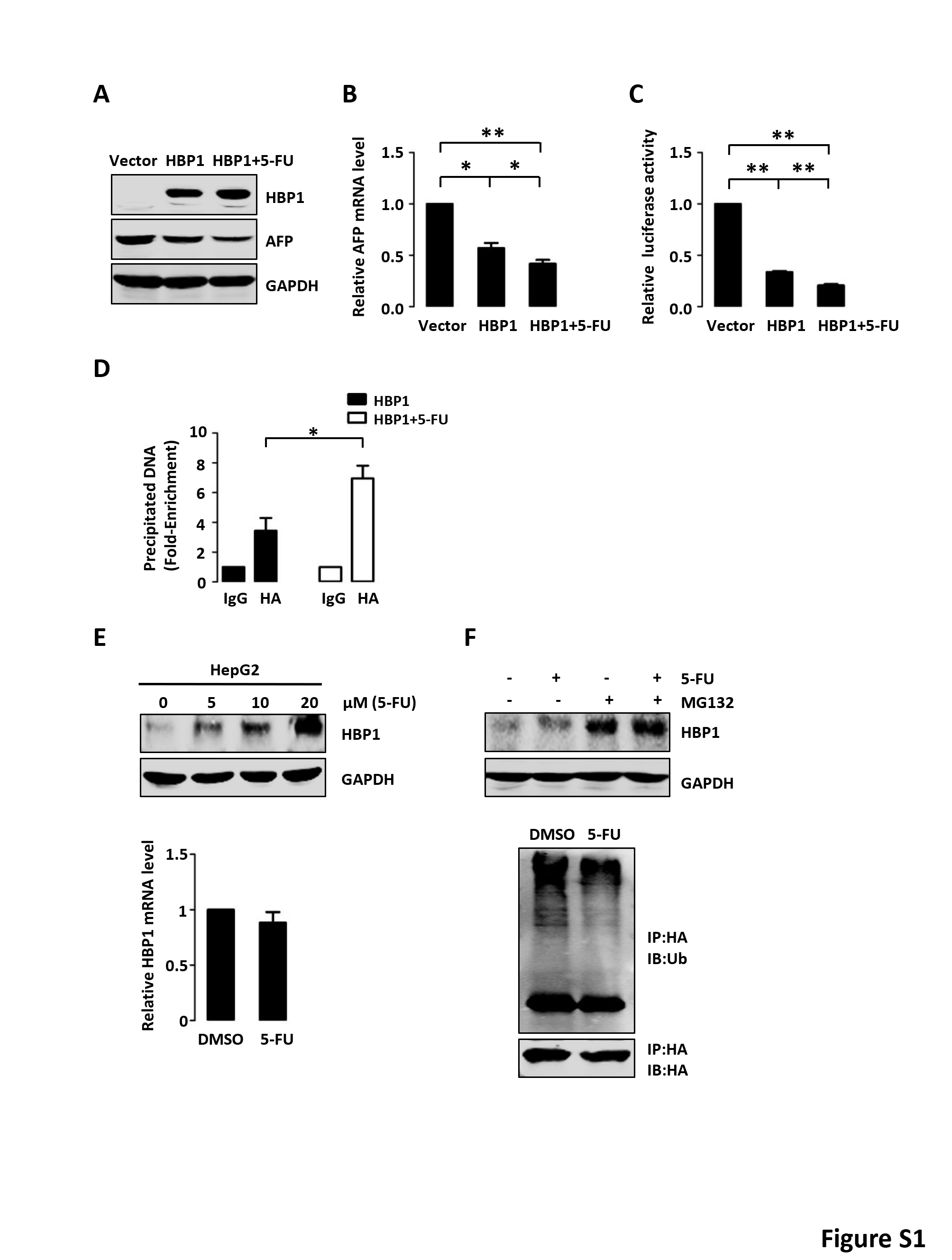

Supplement: Supplementary file 1 — Additional file 1: Figure S1. 5-FU promotes the suppression of HBP1 on AFP through enhancing HBP1 binding to AFP promoter. A-C. 5-FU promotes the suppression of HBP1 on AFP protein (A), mRNA (B) and promoter (C). HepG2 cells were transfected HBP1 with or without 5-FU treatment. The protein levels of HBP1 and AFP were measured by Western blotting. The mRNA level of AFP was measured by Realtime-PCR. The luciferase activities were detected in 293 T cells co-transfected with AFP promoter and HBP1 with or without 5-FU treatment and expressed as the means ± S.D. of the means from three experiments. (D) 5-FU promotes the interaction between HBP1 and AFP promoter. Two hundred ninety-three T cells were transfected HA-HBP1 with or without 5-FU treatment. The region from position − 1600 to position − 1448 contains the HBP1 affinity site and was analyzed by specific PCR. (E) HBP1 protein level is elevated in the presence of 5-FU. HepG2 cells were treated with different concentrations of 5-FU. The protein level was measured by Western blotting (top panel). The mRNA level was measured by Realtime-PCR (bottom panel). (F) 5-FU inhibits HBP1 ubiquitination-mediated proteasome degradation. HepG2 cells were treated with 5-FU with or without MG132. The protein level was measured by Western blotting (top panel). Two hundred ninety-three T cells were transfected HA-HBP1 with or without 5-FU treatment for 24 h and then exposed to MG132 for another 6 h prior to lysis. HBP1 protein was isolated by immunoprecipitation and analyzed by anti-Ub antibody (bottom panel). (G) 5-FU enhances the effect of HBP1 on PTEN, caspase-3, and MMP9 protein levels. HepG2 cells transfected with HBP1 were treated with or without 5-FU. The protein levels were measured by Western blotting. (H) 5-FU enhances HBP1-mediated decrease of cell proliferation. MTT assay was conducted with HepG2 cells stably transfected with control vector, HBP1 with or without 5-FU treatment. Error bars represent S.D. *, p < 0.05, **, p < [file 13046_2021_1881_MOESM1_ESM.zip › Figure S1-1.tif]

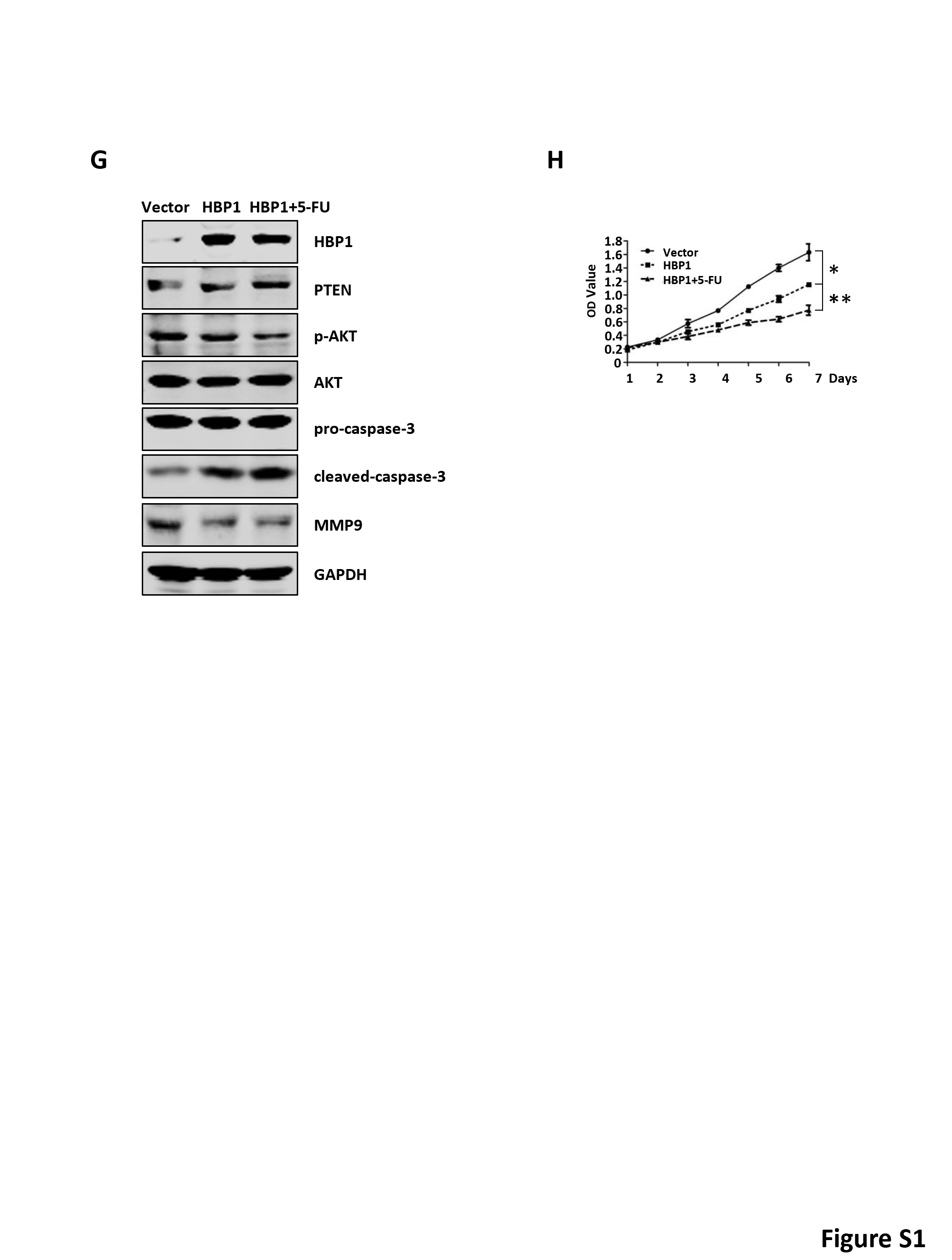

Supplement: Supplementary file 1 — Additional file 1: Figure S1. 5-FU promotes the suppression of HBP1 on AFP through enhancing HBP1 binding to AFP promoter. A-C. 5-FU promotes the suppression of HBP1 on AFP protein (A), mRNA (B) and promoter (C). HepG2 cells were transfected HBP1 with or without 5-FU treatment. The protein levels of HBP1 and AFP were measured by Western blotting. The mRNA level of AFP was measured by Realtime-PCR. The luciferase activities were detected in 293 T cells co-transfected with AFP promoter and HBP1 with or without 5-FU treatment and expressed as the means ± S.D. of the means from three experiments. (D) 5-FU promotes the interaction between HBP1 and AFP promoter. Two hundred ninety-three T cells were transfected HA-HBP1 with or without 5-FU treatment. The region from position − 1600 to position − 1448 contains the HBP1 affinity site and was analyzed by specific PCR. (E) HBP1 protein level is elevated in the presence of 5-FU. HepG2 cells were treated with different concentrations of 5-FU. The protein level was measured by Western blotting (top panel). The mRNA level was measured by Realtime-PCR (bottom panel). (F) 5-FU inhibits HBP1 ubiquitination-mediated proteasome degradation. HepG2 cells were treated with 5-FU with or without MG132. The protein level was measured by Western blotting (top panel). Two hundred ninety-three T cells were transfected HA-HBP1 with or without 5-FU treatment for 24 h and then exposed to MG132 for another 6 h prior to lysis. HBP1 protein was isolated by immunoprecipitation and analyzed by anti-Ub antibody (bottom panel). (G) 5-FU enhances the effect of HBP1 on PTEN, caspase-3, and MMP9 protein levels. HepG2 cells transfected with HBP1 were treated with or without 5-FU. The protein levels were measured by Western blotting. (H) 5-FU enhances HBP1-mediated decrease of cell proliferation. MTT assay was conducted with HepG2 cells stably transfected with control vector, HBP1 with or without 5-FU treatment. Error bars represent S.D. *, p < 0.05, **, p < [file 13046_2021_1881_MOESM1_ESM.zip › Figure S1-2.tif]

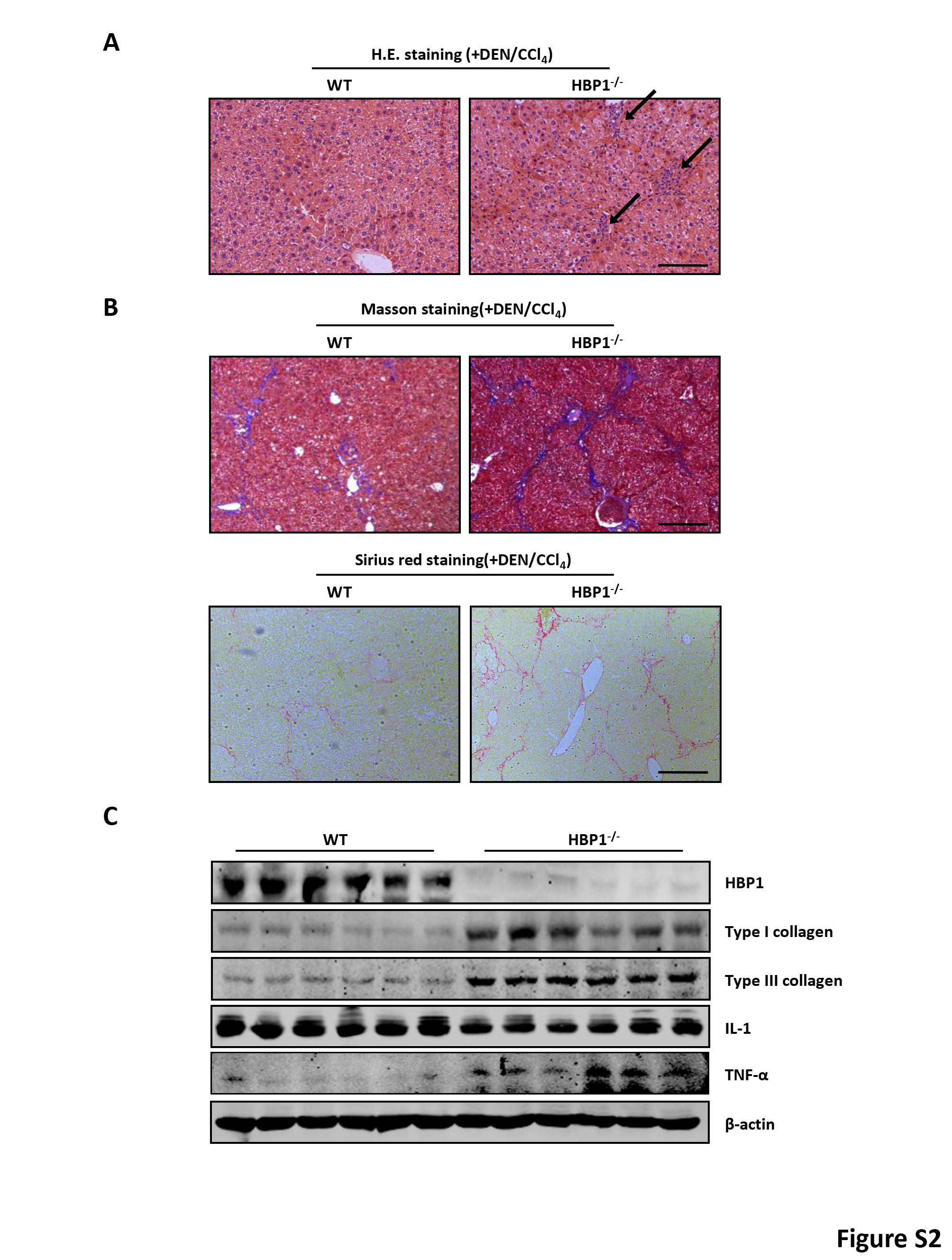

Supplement: Supplementary file 2 — Additional file 2: Figure S2. HBP1 deletion aggravates DEN/CCl4-induced hepatic fibrosis. (A) Representative H.E. staining of liver sections from 12 weeks after DEN/CCl4 treatment in wild type or HBP1−/− mice. Scale bar, 100 μm. (B) Representative images of Masson’s trichrome staining and Sirius red staining of liver sections from the mice described in Figure S2A.. Scale bar, 200 μm. (C) The protein levels of HBP1, TypeIcollagen, Type III collagen, IL-1 and TNF-α in the mice liver described in Figure S2A. were measured by Western blotting. β-actin was used as a loading control. (D) The mRNA levels of TypeIcollagen, Type III collagen, IL-1 and TNF-α were measured by Realtime-PCR in the mice liver described in Figure S2A. (E) Serum ALT and AST levels of the mice described in Figure S2A. The means ± S.D. are shown (n = 5). Error bars represent S.D. *, p < 0.05, **, p < 0.01. [file 13046_2021_1881_MOESM2_ESM.zip › Figure S2-1.tif]

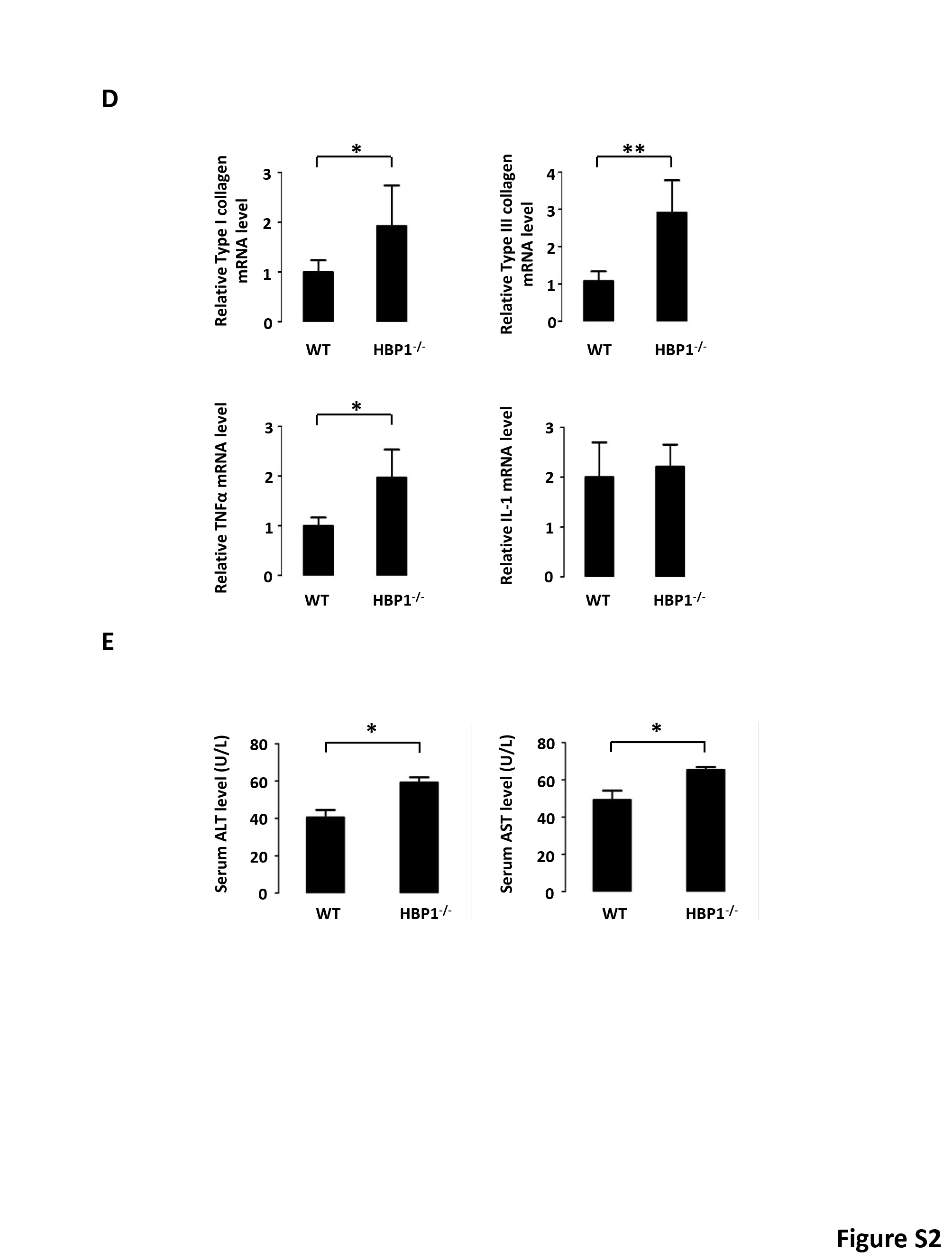

Supplement: Supplementary file 2 — Additional file 2: Figure S2. HBP1 deletion aggravates DEN/CCl4-induced hepatic fibrosis. (A) Representative H.E. staining of liver sections from 12 weeks after DEN/CCl4 treatment in wild type or HBP1−/− mice. Scale bar, 100 μm. (B) Representative images of Masson’s trichrome staining and Sirius red staining of liver sections from the mice described in Figure S2A.. Scale bar, 200 μm. (C) The protein levels of HBP1, TypeIcollagen, Type III collagen, IL-1 and TNF-α in the mice liver described in Figure S2A. were measured by Western blotting. β-actin was used as a loading control. (D) The mRNA levels of TypeIcollagen, Type III collagen, IL-1 and TNF-α were measured by Realtime-PCR in the mice liver described in Figure S2A. (E) Serum ALT and AST levels of the mice described in Figure S2A. The means ± S.D. are shown (n = 5). Error bars represent S.D. *, p < 0.05, **, p < 0.01. [file 13046_2021_1881_MOESM2_ESM.zip › Figure S2-2.tif]

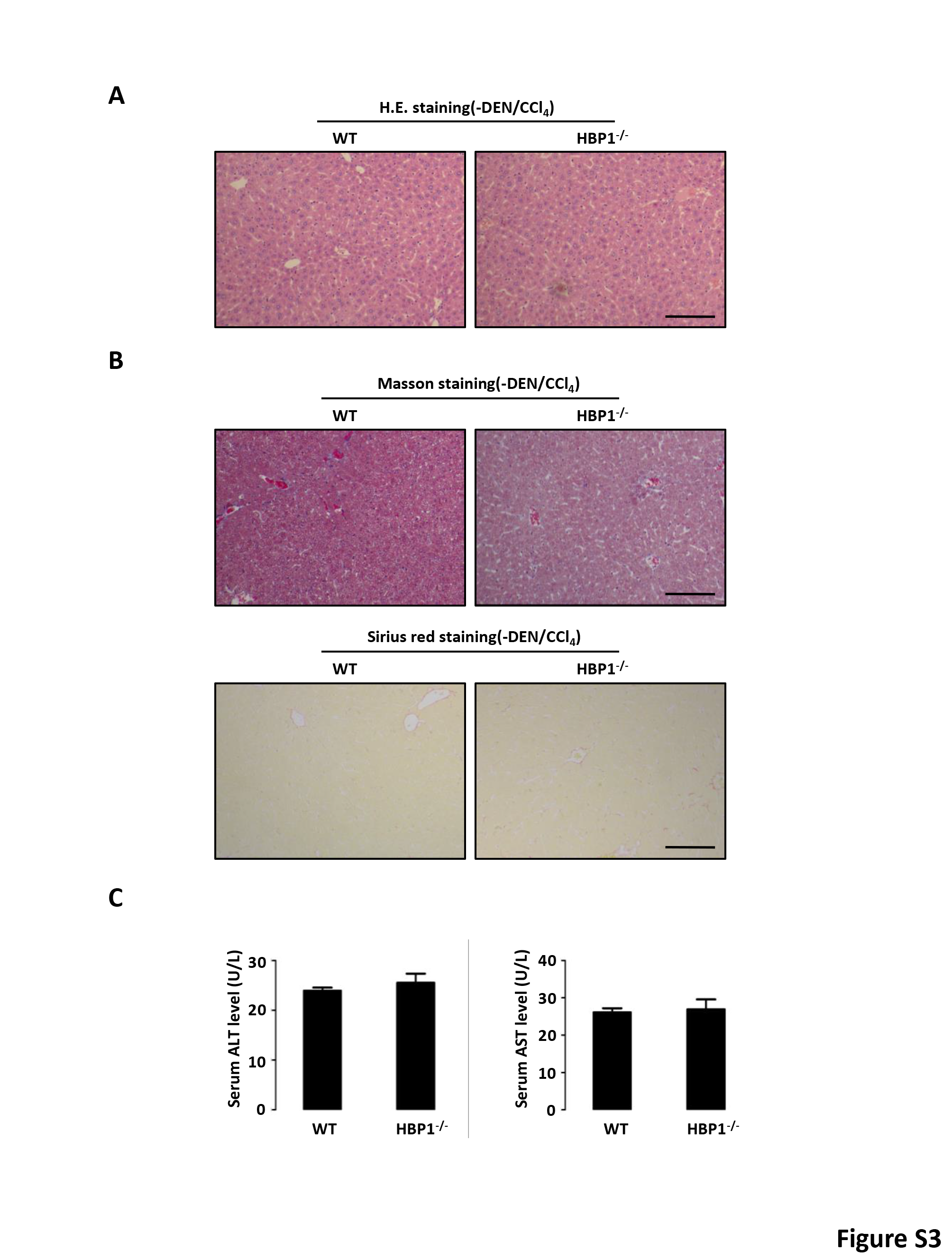

Supplement: Supplementary file 3 — Additional file 3: Figure S3. HBP1 deletion has no effect on liver morphology and function in mice without DEN/CCl4 treatment. (A) Representative H.E. staining of liver sections from 12 weeks after saline treatment in wild type or HBP1−/− mice. Scale bar, 100 μm. (B) Representative images of Masson’s trichrome staining and Sirius red staining of liver sections from the mice described in Figure S3A. Scale bar, 200 μm. (C) Serum ALT and AST levels of the mice described in Figure S3A. The means ± S.D. are shown (n = 5). Error bars represent S.D. [file 13046_2021_1881_MOESM3_ESM.tif]

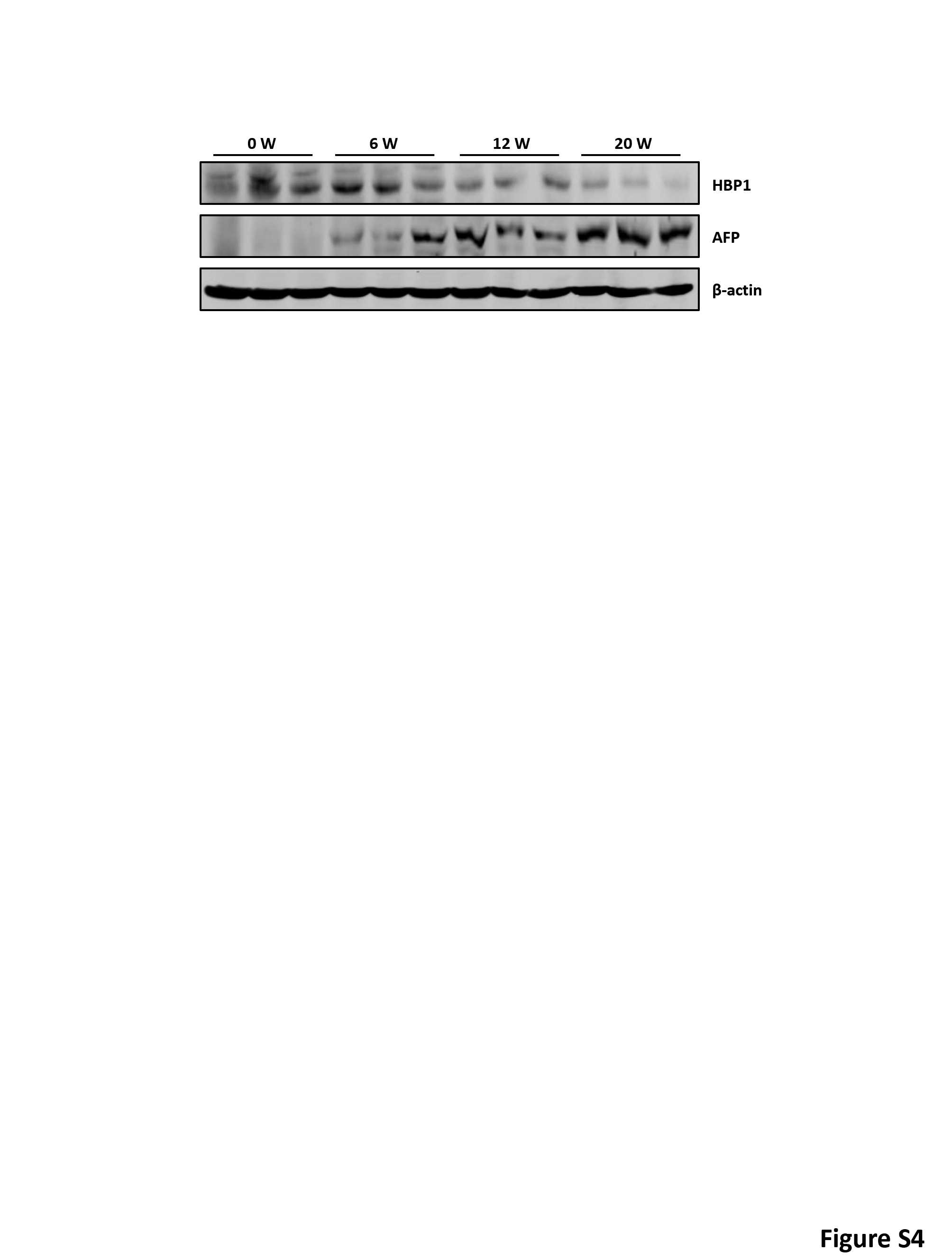

Supplement: Supplementary file 4 — Additional file 4: Figure S4. There is a negative correlation between the expression of HBP1 and AFP in DEN/CCl4-induced hepatoma in mice. The protein levels of HBP1 and AFP in the liver were measured by Western blotting during DEN/CCl4 induction. β-actin was used as a loading control. [file 13046_2021_1881_MOESM4_ESM.tif]
